# Supplementary material for: Stromal Cell-Contact Dependent PI3K and APRIL Induced NF-κB Signaling Prevent Mitochondrial- and ER Stress Induced Death of Memory Plasma Cells
Source: Cell Rep. 2020 Aug 4;32(5):107982. doi: 10.1016/j.celrep.2020.107982 (PMC7408492; doi:10.1016/j.celrep.2020.107982)
Supplement: Document S1. Figures S1–S4 [file mmc1.pdf]

Cell Reports, Volume *32*

Supplemental Information

Stromal Cell-Contact Dependent PI3K and APRIL Induced NF-κB Signaling Prevent Mitochondrial- and ER Stress Induced Death of Memory Plasma Cells

Rebecca Cornelis, Stefanie Hahne, Adriano Taddeo, Georg Petkau, Darya Malko, Pawel Durek, Manja Thiem, Lukas Heiberger, Lena Peter, Elodie Mohr, Cora Klaeden, Koji Tokoyoda, Francesco Siracusa, Bimba Franziska Hoyer, Falk Hiepe, Mir-Farzin Mashreghi, Fritz Melchers, Hyun-Dong Chang, and Andreas Radbruch

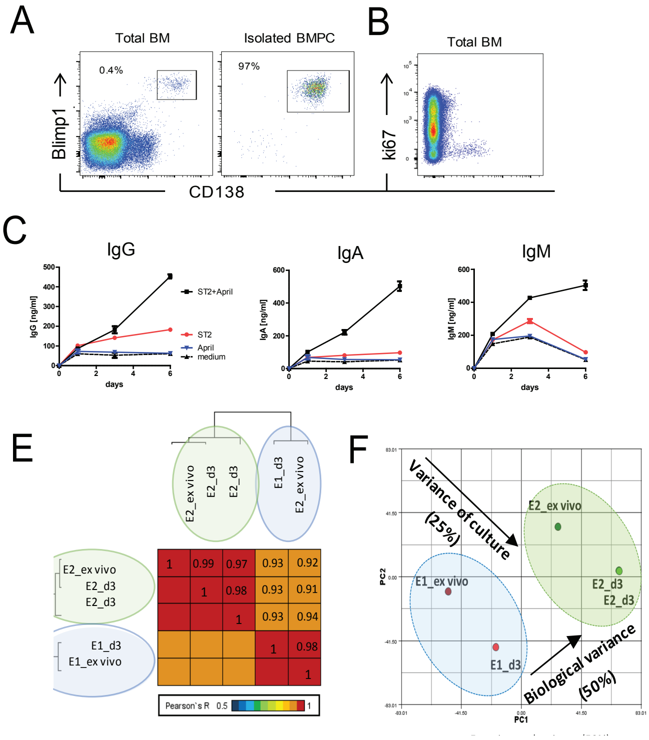

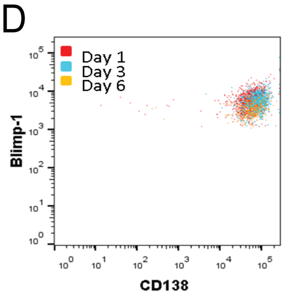

G

Symbol	mean ex vivo	mean d3	FC
Prdm1/Blimp-1	3864.665	3035.403	0.78
Sdc1/CD138	3339.13	3278.153	0.98
Bcl2	1347.885	1027.57	0.76
Bcl2l11/Bim	1051.34	1206.74	1.14
Pmaip1/Noxa	333.795	371.78	1.11
Mcl1	1928.82	2171.1	1.12
Irf4	5933.32	6511.95	1.09
Foxo1	731.06	937.07	1.28
Foxo3	2421.92	2544.27	1.05

H

	Affy_ID	Symbol	UP:Protein_Name	Limma_q	Mean_Ex vivo plasma cells	Mean_cultured plasma cells	fold-change
Group A	1423100_at	Fos	Proto-oncogene c-Fos;	1.49E-03	8,917.44	*not present*	
	1422134_at	Fosb	Protein fosB;	1.49E-03	4,600.63	*not present*	
	1417394_at	Klf4	Krueppel-like factor 4;	3.71E-02	372.06	*not present*	
	1418243_at	Fcna	Ficolin-1;	3.75E-02	252.85	*not present*	
	1420166_at	Dntt	DNA nucleo dylexotransferase;	4.71E-02	144.31	*not present*	
	1417063_at	C1qb	Complement C1q subcomponent subunit B;	3.71E-02	129.39	*not present*	
	1417409_at	Jun	Transcrip on factor AP-1;	3.71E-02	4,521.24	185.9	-24
	1448830_at	Dusp1	Dual specificity protein phosphatase 1;	3.26E-03	2,884.55	140.41	-21
	1448694_at	Jun	Transcrip on factor AP-1;	1.46E-02	2,537.95	205.33	-12
	1457404_at	Nfkbiz	NF-kappa-B inhibitor zeta;	3.84E-02	580.27	116.43	-5
	1430979_a_at	Prdx2	Peroxiredoxin-2;	4.71E-02	1,103.37	355.27	-3
	1424854_at	Hist1h4i	Histone H4;	3.75E-02	295.92	115.42	-3
	1422632_at	Ctsw	Cathepsin W;	4.71E-02	982.67	400.7	-2
Group B	1415918_a_at	Tpi1	Triosephosphate isomerase;	3.71E-02	400.97	3,633.08	9
	1452927_x_at	Tpi1	Triosephosphate isomerase;	3.71E-02	518.91	3,964.05	8
	1419030_at	Ero1l	ERO1-like protein alpha;	3.80E-02	372.59	1,884.26	5
	1433930_at	Hpse	Heparanase;	3.71E-02	231.81	1,102.99	5
	1451461_a_at	Aldoc	Fructose-bisphosphate aldolase C;	3.71E-02	276.27	1,212.16	4
	1422470_at	Bnip3	BCL2/adenovirus E1B 19 kDa protein-interac ng protein 3;	3.71E-02	224.54	954.42	4
	1439148_a_at	Pfkl	ATP-dependent 6-phosphofructokinase, liver type ;	3.75E-02	390.51	1,579.83	4
	1449324_at	Ero1l	ERO1-like protein alpha;	4.71E-02	651.93	2,260.04	3
	1421057_at	Dnase1l3	Deoxyribonuclease gamma;	3.71E-02	183.88	616.78	3
	1452094_at	P4ha1	Prolyl 4-hydroxylase subunit alpha-1;	4.71E-02	361.28	1,168.42	3
	1451895_a_at	Dusp9	Dual specificity protein phosphatase ;	3.71E-02	319.3	951.66	3
	1417864_at	Pgk1	Phosphoglycerate kinase 1;	4.71E-02	2,606.08	7,562.93	3
	1433604_x_at	Aldoa	Fructose-bisphosphate aldolase;	3.71E-02	2,974.82	8,013.74	3
	1418129_at	Dhcr24	Delta(24)-sterol reductase;	4.71E-02	332.98	837.46	3
	1429781_s_at	Ccdc39	Coiled-coil domain-containing protein 39;	4.30E-02	247.12	586.02	2
Group C	1448823_at	Cxcl12	Stromal cell-derived factor 1;	4.26E-02	*not present*	808.34	
	1438945_x_at	Gja1	Gap junc on alpha-1 protein;	3.71E-02	*not present*	453.34	
	1448433_a_at	Pcolce	Procollagen C-endopep dase enhancer 1;	3.71E-02	*not present*	383.29	
	1452250_a_at	Col6a2	Collagen alpha-2(VI) chain;	3.71E-02	*not present*	342.52	
	1424770_at	Cald1	H-caldesmon ;	4.26E-02	*not present*	272.95	
	1434679_at	Ncan	Neurocan core protein;	4.71E-02	*not present*	246.65	
	1449145_a_at	Cav1	Caveolin-1;	2.14E-02	*not present*	130.35	
	1448494_at	Gas1	Growth arrest-specific protein 1;	4.12E-02	*not present*	121.73	
	1425028_a_at	Tpm2	Tropomyosin beta chain;	3.75E-02	*not present*	120.12	
	1416686_at	Plod2	Procollagen-lysine,2-oxoglutarate 5-dioxygenase 2;	4.71E-02	*not present*	101.73	
	1424114_s_at	Lamb1	Laminin subunit beta-1;	3.80E-02	*not present*	71.56	
	1429479_at	Magef1	Puta ve uncharacterized protein ;	4.12E-02	*not present*	51.38	

S1. Bone marrow memory plasma cells maintain their transcriptional profile and remain functional during in vitro culture. Related to Figure 1. (A) Original fraction of bone marrow from immunized C57BL/6J mice containing 0.4% of CD138++ plasma cells and purity of isolated plasma cells co-expressing BLIMP-1. (B) Ki-67 versus CD138 staining of total bone marrow. (C) Quantification of IgG, IgA and IgM in the supernatant of cultured plasma cells taken at the indicated time points by ELISA (n=1). (D) Expression of CD138 and BLIMP-1 in plasma cells cultured with ST2 in the presence of APRIL and measured on day 1, 3 and 6 of culture. (E) Pearson correlation of the global transcriptome of ex vivo isolated and 3 days cultured plasma cells. (F) Principal component analysis of two individual gene expression analyses of plasma cells ex vivo isolated and after 3 day culture. (G) Mean expression and fold-change of plasma cell specific genes and survival genes in ex vivo and 3 days cultured plasma cells. (H) Transcriptomes of memory plasma cells ex vivo and after 3 days of culture were analyzed for differentially expressed genes. The table shows statistically differentially expressed genes with adjusted p-value < 0.05 and the corresponding mean expression values.

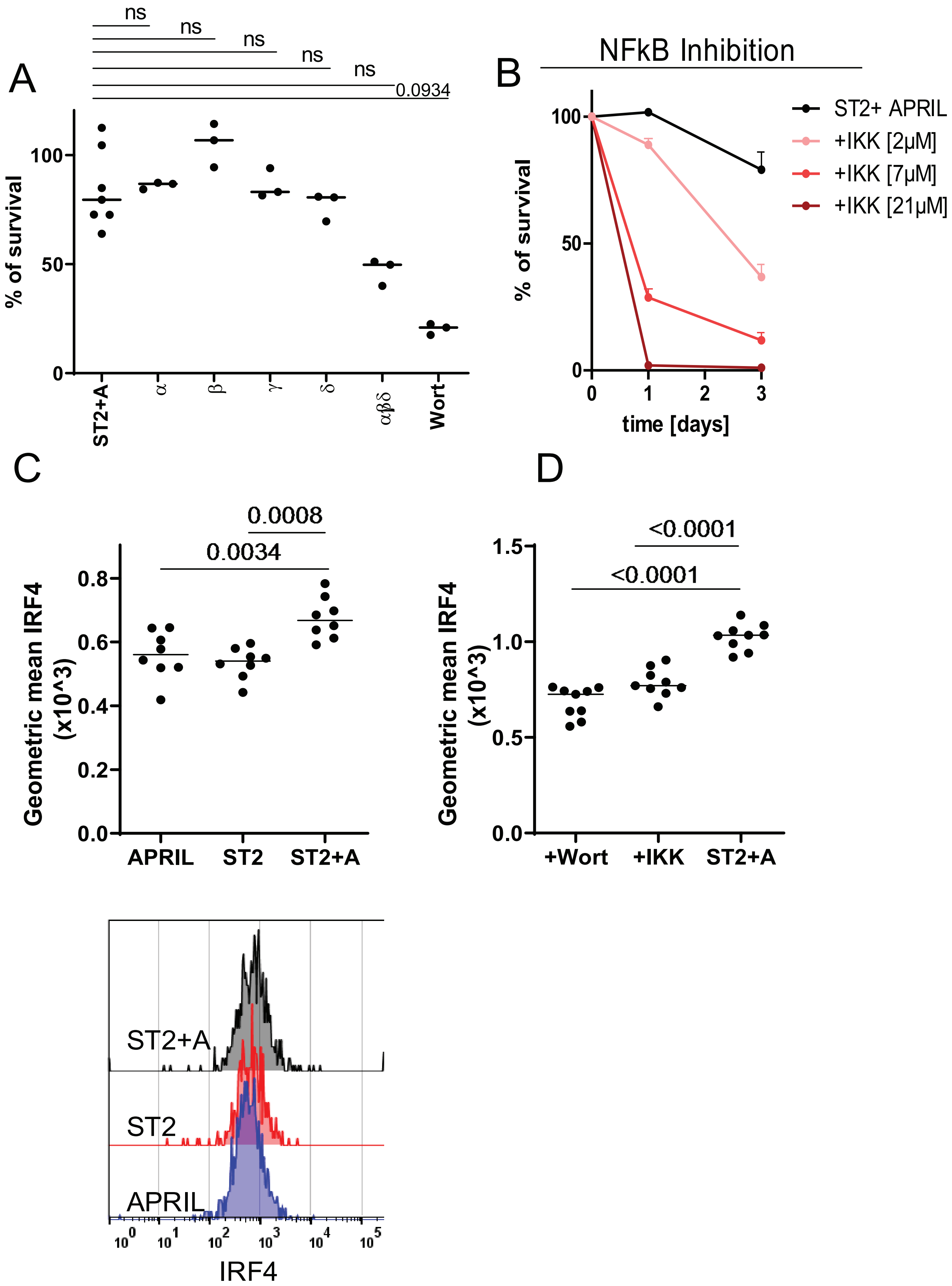

S2. Memory plasma cell survival and IRF4 induction depends on NF-kB and PI3K signaling. Related to Figure 2.
(A) Survival of memory plasma cells treated with inhibitors against different subunits of PI3K or Wortmannin as control (technical replicates n=3-6). Cells were enumerated by flow cytometry on day 1 of culture. Statistics: Kruskal-Wallis test
(B) Survival of memory plasma cells pre-treated with the irreversible inhibitor IKK-16.
Cells were enumerated by flow cytometry on day 1 and 3 of culture.
(C, D) IRF4 protein expression, shown as geometric mean, in CD138+ plasma cells cultured for 1 day with or without ST2 cells, with or without APRIL or pre-treated with either 10 µM Wortmannin or 2.5 µM of IKK16 (pooled from two independent experiments with technical replicates n=6-8 for each group). Statistics: Ordinary one-way ANOVA.

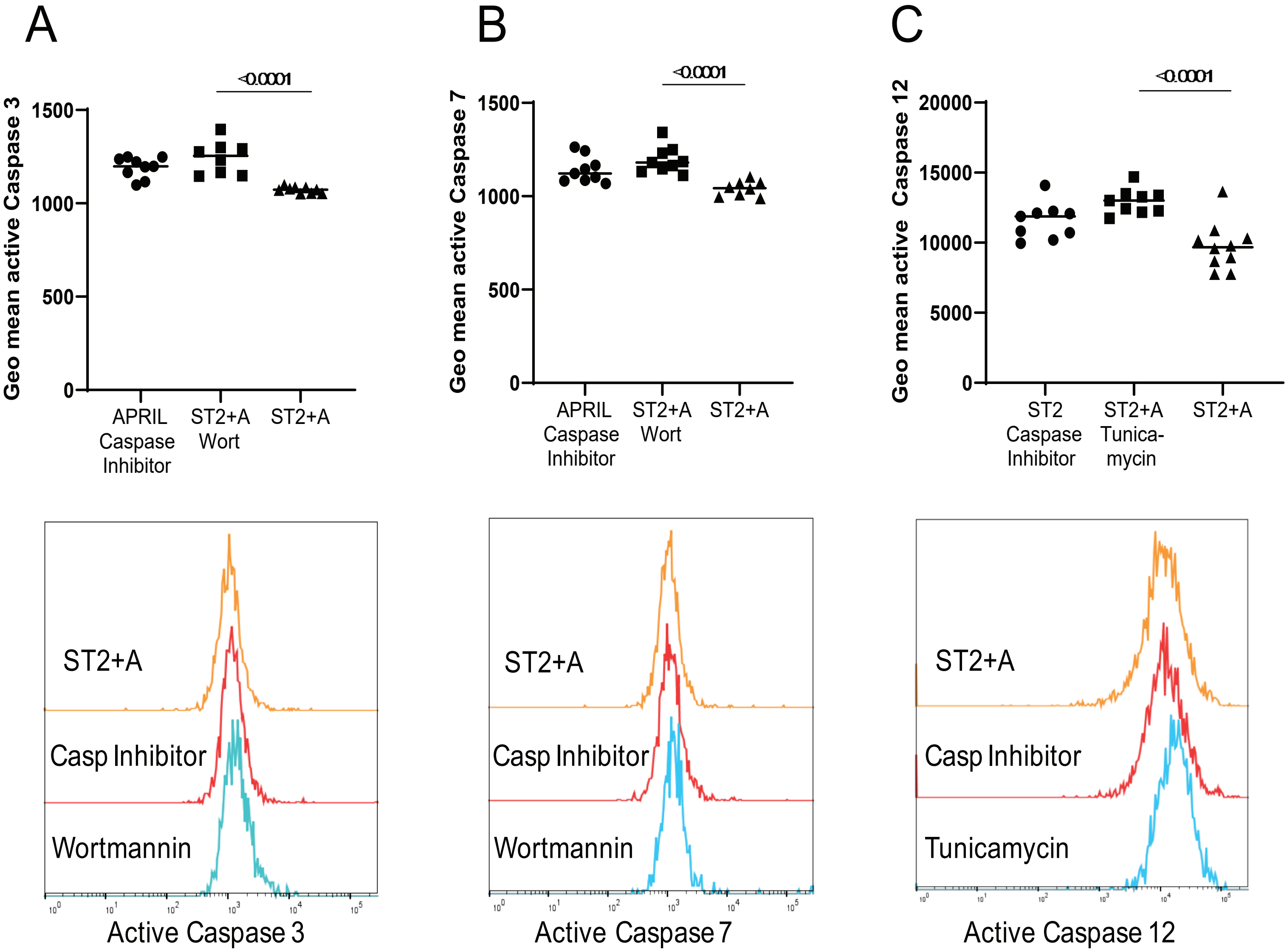

S3. Expression levels of active Caspases 3, 7 and 12 using Wortmannin or tunicamycin as controls. Related to Figure 4.
Expression of active Caspase 3 (A), 7 (B) and 12 (C), shown as geometric mean, in live CD138+ plasma cells cultured for 1 day with APRIL and/or ST2 cells and pan Caspase Inhibitor, and with or without treatment with either 10 µM Wortmannin or 2.5 µM Tunicamcyin for 2 hours (pooled from two independent experiments with technical replicates n=9 for each group). Statistics: Ordinary one-way ANOVA.

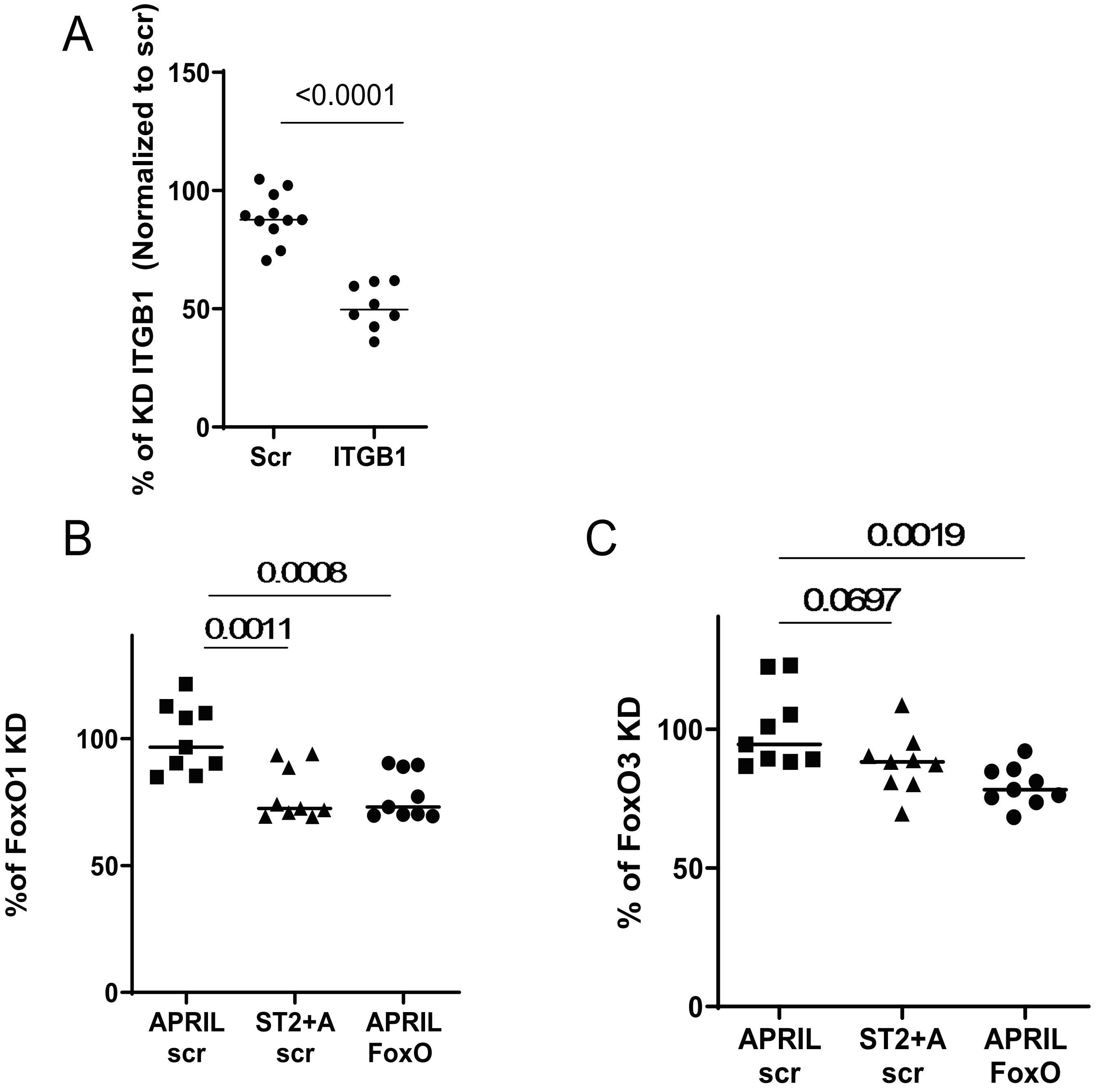

S4. Knock-down efficiency of siRNAs targeting ITGB1, FoxO1 and FoxO3. Related to Figures 1 and 3.
ITGB1 (A), FoxO1 (B) and FoxO3 (C) protein expression was measured in viable CD138+ plasma cells at day 3 of culture with the indicated siRNAs (pooled from three independent experiments with technical replicates n =9 for each group).
Statistics: t-test (ITGB1), ordinary one-way ANOVA (FoxO1 and FoxO3).
