# Supplementary material for: Stromal Cell-Contact Dependent PI3K and APRIL Induced NF-κB Signaling Prevent Mitochondrial- and ER Stress Induced Death of Memory Plasma Cells
Source: Cell Rep. 2020 Aug 4;32(5):107982. doi: 10.1016/j.celrep.2020.107982 (PMC7408492; doi:10.1016/j.celrep.2020.107982)
Supplement: Document S2. Article plus Supplemental Information [file mmc2.pdf]

Cell Reports

Report

# Stromal Cell-Contact Dependent PI3K and APRIL Induced NF-κB Signaling Prevent Mitochondrial- and ER Stress Induced Death of Memory Plasma Cells

## Graphical Abstract

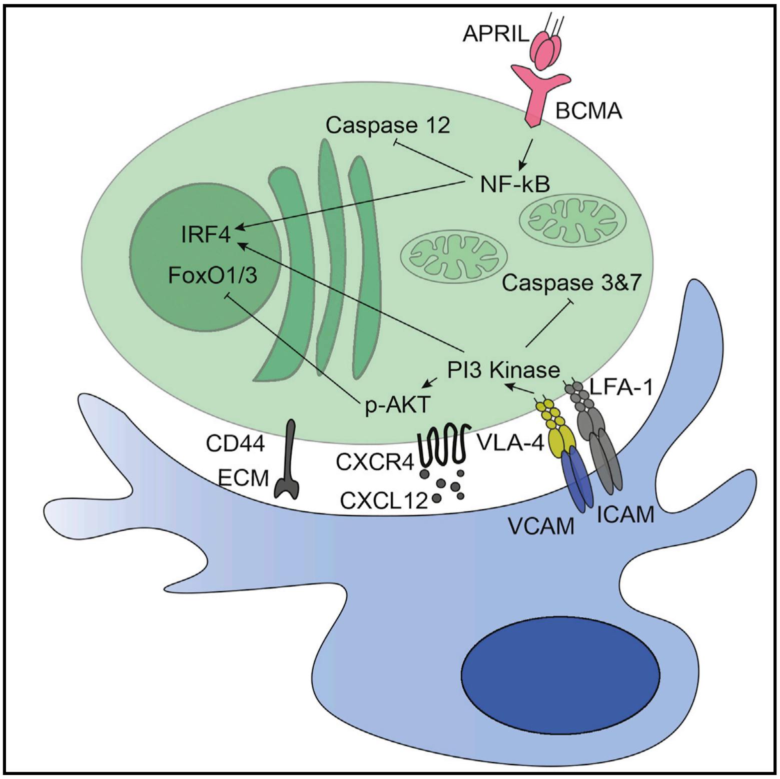


## Highlights

- Description of an *in vitro* niche for the cultivation of primary murine plasma cells
- Stromal cell contact and APRIL are essential for survival of bone marrow plasma cells
- Stromal-cell-contact-induced PI3K signaling inhibits FoxO1/3 and caspase 3 activation
- APRIL-induced NF-κB signaling prevents caspase 12 activation


## Authors

Rebecca Cornelis, Stefanie Hahne, Adriano Taddeo, ..., Fritz Melchers, Hyun-Dong Chang, Andreas Radbruch

## Correspondence

radbruch@drfz.de



## In Brief

In this study, Cornelis et al. address the molecular mechanisms underlying the survival of murine memory plasma cells in the bone marrow. The authors provide evidence that direct contact to stromal cells and exogenous APRIL provide resilience to mitochondrial and endoplasmic stress, respectively, synergistically promoting plasma cell survival.


Cornelis et al., 2020, Cell Reports *32*, 107982
August 4, 2020 © 2020 The Authors.
https://doi.org/10.1016/j.celrep.2020.107982

CellPress

Cell Reports

CellPress
OPEN ACCESS

Report

# Stromal Cell-Contact Dependent PI3K and APRIL Induced NF-κB Signaling Prevent Mitochondrial- and ER Stress Induced Death of Memory Plasma Cells

Rebecca Cornelis,[1,3] Stefanie Hahne,[1,3] Adriano Taddeo,[1,5] Georg Petkau,[1] Darya Malko,[1] Pawel Durek,[1] Manja Thiem,[1] Lukas Heiberger,[1] Lena Peter,[1] Elodie Mohr,[1] Cora Klaeden,[1] Koji Tokoyoda,[1] Francesco Siracusa,[1,6] Bimba Franziska Hoyer,[1,2] Falk Hiepe,[1,2] Mir-Farzin Mashreghi,[1] Fritz Melchers,[1] Hyun-Dong Chang,[1,4] and Andreas Radbruch[1,4,7,*]

[1]Deutsches Rheuma-Forschungszentrum Berlin (DRFZ), a Leibniz Institute, Chariteplatz1, 10117 Berlin, Germany
[2]Charité-Universitätsmedizin Berlin, Department of Rheumatology and Clinical Immunology, 10117 Berlin, Germany
[3]These authors contributed equally
[4]These authors contributed equally
[5]Present address: Institute of Life Technologies, University of Applied Sciences and Arts Western Switzerland, Delémont, Switzerland
[6]Present address: Department of General, Visceral and Thoracic Surgery, University Medical Center Hamburg-Eppendorf, Hamburg, Germany
[7]Lead Contact
*Correspondence: radbruch@drfz.de
https://doi.org/10.1016/j.celrep.2020.107982

## SUMMARY

The persistence of long-lived memory plasma cells in the bone marrow depends on survival factors available in the bone marrow, which are provided in niches organized by stromal cells. Using an *ex vivo* system in which we supply the known survival signals, direct cell contact to stromal cells, and the soluble cytokine a proliferation-inducing ligand (APRIL), we have elucidated the critical signaling pathways required for the survival of long-lived plasma cells. Integrin-mediated contact of bone marrow plasma cells with stromal cells activates the phosphatidylinositol 3-kinase (PI3K) signaling pathway, leading to critical inactivation of Forkhead-Box-Protein O1/3 (FoxO1/3) and preventing the activation of mitochondrial stress-associated effector caspases 3 and 7. Accordingly, inhibition of PI3K signaling *in vivo* ablates bone marrow plasma cells. APRIL signaling, by the nuclear factor κB (NF-κB) pathway, blocks activation of the endoplasmic-reticulum-stress-associated initiator caspase 12. Thus, stromal-cell-contact-induced PI3K and APRIL-induced NF-κB signaling provide the necessary and complementary signals to maintain bone marrow memory plasma cells.

## INTRODUCTION

Plasma cells (PCs) can persist for long time periods in the bone marrow (BM). However, PCs are not intrinsically long lived (Makela and Nossal, 1962; Schooley, 1961) and die quickly when isolated and cultured *in vitro*, suggesting that their persistence in the BM depends on survival factors provided in the BM (Cassese et al., 2003). In the BM, PCs are located individually in direct contact to mesenchymal stromal cells, and it has been postulated that these stromal cells organize a survival niche for the PCs (Manz and Radbruch, 2002; Radbruch et al., 2006; Tokoyoda et al., 2004; Zehentmeier et al., 2014). How the survival is mediated at a molecular level has remained unclear. Essential for PC survival is signaling through the B cell maturation antigen (BCMA; CD269) receptor of the PCs (O'Connor et al., 2004) induced by its two ligands, namely, a proliferation-inducing ligand (APRIL; CD256) or B-cell-activating factor (BAFF; BLys and CD257) (Benson et al., 2008) and the antiapoptotic protein myeloid cell leukemia 1 (MCL-1) (Peperzak et al., 2013). Evidence suggests that stromal cells might contribute directly to PC survival in the BM. Antibodies against the adhesion molecules integrin αLβ2 (lymphocyte-function-associated antigen [LFA-1]; CD11a/CD18) and integrin α4β1 (very late antigen [VLA-4]; CD49d/CD29), expressed by the PCs, ablate PCs from the BM (DiLillo et al., 2008). Ligands for both of these integrins, vascular cell adhesion molecule 1 (VCAM; CD106) and intercellular adhesion molecule 1 (ICAM; CD54), are expressed by BM stromal cells, suggesting that integrin-mediated binding of PCs to stromal cells might directly, by the focal adhesion kinase/phosphatidylinositol 3-kinase (PI3K) pathway (Giancotti and Ruoslahti 1999; Parsons et al., 2000) promote PC persistence in the BM. In line with this idea, CD37-deficient antibody-secreting cells, which show impaired clustering of VLA-4, have diminished PI3K signaling and impaired survival (Van Spriel et al., 2012). Whether or not BCMA signaling and contact to stromal cells are sufficient to maintain the survival of memory PCs and how they prevent death of the PC have not been elucidated.

Here, we demonstrate, both *in vivo* and *ex vivo*, that BM PC survival is dependent on PI3K signaling. PI3K signaling is induced in PCs by direct contact to stromal cells and leads to


© 2020 The Authors.
This is an open access article under the CC BY license (http://creativecommons.org/licenses/by/4.0/).

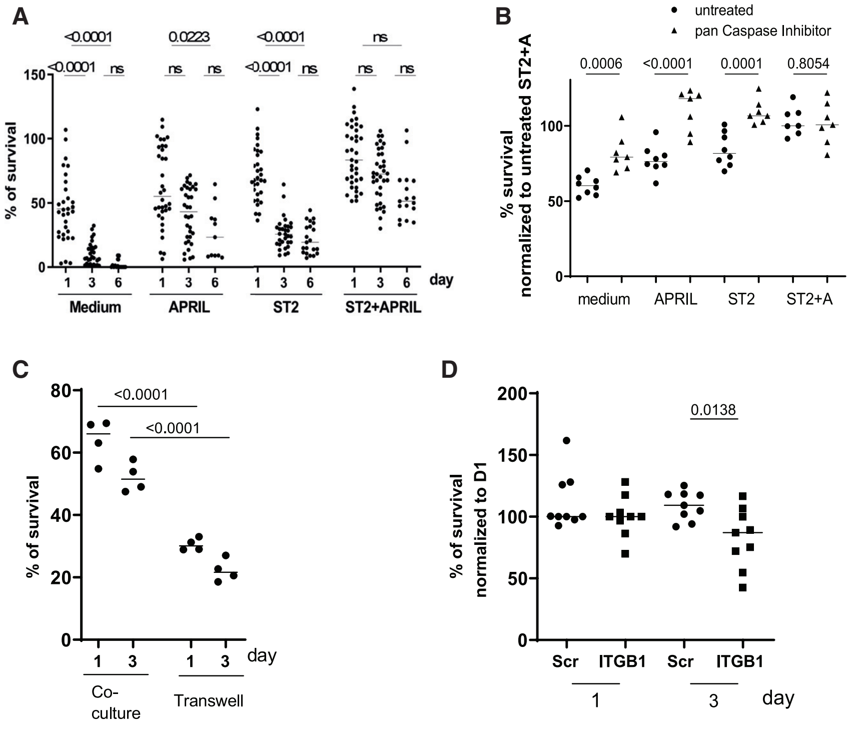


**Figure 1. Survival of Bone Marrow Memory PCs Is Dependent on Direct Cell Contact with Stromal Cells and the Presence of APRIL**

(A) Survival of primary murine bone marrow PCs cultured ± ST2 cells and ± APRIL for up to 6 days at 4.2% $O_2$. Viable plasma cells ($CD138^{++}/DAPI^{-}$) were counted by flow cytometry. Median of at least 5 pooled independent experiments with at least n = 14 technical replicates for each group. Statistics: Kruskal-Wallis test.

(B) Isolated PCs treated with or without pan-caspase inhibitor when cultured ± ST2 cells and ± APRIL. Viable PCs were counted on day 1 of culture (pooled from two independent experiments with a minimum of n = 7 technical replicates for each group). Statistics: ordinary one-way ANOVA.

(C) Survival of PCs in the presence of APRIL on day 1 and day 3, when cultured in transwell or directly contacting ST2 cells (pooled from two independent experiments with n = 4 technical replicates for each group). Statistics: t test.

(D) Survival of PCs on day 1 and day 3 treated with specific siRNA directed against ITGB1 and scrambled controls (pooled from three independent experiments with n = 9 technical replicates for each group). Statistics: ordinary one-way ANOVA.

inactivation of Forkhead-Box-Protein O1/3 (FoxO1/3), which is essential for the survival of the PCs. PC survival also depends on nuclear factor "kappa-light-chain-enhancer" of activated B cell (nuclear factor κB [NF-κB]) signaling, which is induced by APRIL. Pan-caspase inhibition can substitute for both signaling pathways and rescues PC survival *in vitro*. Interestingly, stromal cell contact alone but not APRIL prevents activation of the mitochondrial-stress-associated caspases 3 (Casp3) and 7, whereas APRIL prevents activation of the endoplasmic reticulum (ER)-associated Casp12.

## RESULTS

### Contact to Stromal Cells and APRIL-Induced Signaling Pathways Prevent Caspase-Mediated Cell Death of PCs

Memory PCs were isolated from the BM of chicken gamma globulin (CGG)-immunized C57BL/6J mice more than 30 days after the last immunization by magnetic depletion of $CD49b^{+}$ and $B220^{+}$ cells, followed by magnetic enrichment of $CD138^{+}$ cells. Using this protocol, we achieved purities of more than 90% and recovery rates of about 35% of viable BM PCs. Isolated PCs expressed the PC transcription factor BLIMP-1 and were Ki-67 negative, the latter indicating that the cells were resting in terms of proliferation (Figure S1A and S1B). The cells were cultured *in vitro* with or without murine stromal cell line ST2 at an initial ratio of 1:1 in the presence or absence of APRIL. On days 1, 3, and 6 of the culture, viable PCs ($CD138^{++}$/4′,6-diamidine-2-phenylindole dihydrochloride negative [$DAPI^{-}$]) were enumerated and analyzed by flow cytometry. All cultures were performed under physiological oxygen levels of 4.2% $O_2$ to mimic the BM environment (Nguyen et al., 2018; Spencer et al., 2014). PCs rapidly died within days when isolated from the BM and cultured in medium (median viability: day 1: 43.27%, day 3: 7.095%, day 6: 0%). However, PC survival was significantly improved when the cells were co-cultured with ST2 cells and in the presence of the cytokine APRIL (median viability: day 1, 83.14%; day 3, 72.19%; day 6, 51.20%). Co-culture of PCs with ST2 cells alone (median viability: day 1, 67.47%; day 3, 25.42%; day 6, 19.07%) or with APRIL alone (median viability: day 1, 55.24%; day 3, 43.15%; day 6, 23.27%) were not sufficient to maintain PCs alive (Figure 1A). The expression of CD138 and BLIMP-1 on the PCs was not altered during the 6 days of *in vitro* culture with ST2 cells and APRIL, and antibody secretion was maintained (Figures S1C and S1D). To confirm that the identity of PCs was maintained for 3 days in co-culture with ST2 cells and APRIL, we compared their global transcriptomes to those of *ex-vivo*-isolated PCs (Figures S1E and S1F). Of 10,000 genes expressed at statistically significant levels, only 41 genes showed a significant difference ($p < 0.01$) in expression before and after culture (Figure S1H). The transcription factor AP-1 (JunB, Jun, Fos) was highly expressed in PCs isolated from BM but was not or only marginally expressed in PCs cultivated for 3 days. Expression of AP-1 and other stress-inducible genes (12; group A) may reflect stress induced by the tedious isolation procedure of PCs from the BM, as compared to their isolation from cell culture. A number of hypoxia-related and metabolic genes (15 genes; group B) were upregulated in cultivated PCs, compared with PCs directly isolated from the BM. This finding may reflect the extended time the PCs spent under normoxic conditions during the isolation period. Finally, other genes (12 genes, group C), most prominently CXCL12, not expressed in PCs isolated directly from the

BM, but in those isolated from cell culture on day 3, may indicate contaminating stromal cells that were abundant in cell culture due to the ST2 cell line. Expression of the genes *CD138*, *Foxo1* and *3*, *Prdm1*, *Irf4*, *Noxa*, *Bcl2l11*, *Bcl2*, and *Mcl1* was not significantly different (Figure S1G).

The survival of *ex-vivo*-isolated BM PCs cultured with APRIL or ST2 cells alone was rescued by pan-caspase inhibitors (Figure 1B), suggesting that co-culture of PCs with ST2 cells and APRIL prevents caspase-mediated cell death.

Interaction between stromal cells and PCs has been suggested to be mediated by direct cell contact (Manz and Radbruch, 2002; Radbruch et al., 2006; Tokoyoda et al., 2004; Zehentmeier et al., 2014) by VCAM1/VLA4 and ICAM/LFA1 interaction (DiLillo et al., 2008). When culturing PCs and ST2 cells in a transwell assay, survival of PCs was significantly decreased (median viability for ST2 cells: day 1, 66%; day 3, 51%; and for PCs: day 1, 30%; day 3, 22%) compared with co-culturing conditions (Figure 1C). Small interfering RNA (siRNA)-mediated knockdown of integrin β1 (CD29/ITGB1), a subunit of the VLA4 (Integrin a4β1) heterodimer by 50% (Figure S4A), significantly reduced the survival of PCs in co-culture with ST2 cells and APRIL *ex vivo* (Figure 1D), indicating that direct cell contact is required for survival and that contact-mediated survival is in part mediated by integrin β1 (median viability for scrambeld (scr): day 1, 100%; day 3, 109%; and for ITGB1: day 1, 100%; day 3, 87%).

### Inhibition of PI3K Signaling Results in PC Death *Ex Vivo*

Preincubation of *ex-vivo*-isolated BM PCs with the irreversible PI3K inhibitor Wortmannin resulted in a dose-dependent decrease in survival of the PCs, when co-cultured with ST2 cells in the presence of APRIL (viability for ST2+A: day 1, 101%; day 3, 76%; +Wortmannin 0.6 μM: day 1, 100%; day 3, 66%; +Wortmannin 3 μM: day 1, 102%; day 3, 66%; +Wortmannin 15 μM: day 1, 73%; day 3, 37%; +Wortmannin 77 μM: day 1, 68%; day 3, 20%) (Figure 2A). Using an alternative pan-PI3K inhibitor, LY294002, PC survival was also decreased in a dose-dependent manner (viability of ST2+A +LY29400 10 μM: day 1, 62%; day 3, 68%; +LY29400 20 μM: day 1, 44%; day 3, 37%; +LY29400 40 μM, day 1, 24%; day 3, 5.7%) (Figure 2B). More specific inhibition of any of the four known PI3K subunits α, β, γ, or δ did not impact the survival of PC (Figure S2A). Only when any three subunits were simultaneously inhibited, PC survival was reduced to a similar degree as it was observed in the presence of Wortmannin (Figure 2C). Apparently, PCs have no particular requirement regarding the subunit composition of their PI3K. Inhibition of the NF-κB pathway downstream of BCMA, the receptor for APRIL, with the pan-NF-κB inhibitor IKK16 (Thein et al., 2014; Waelchli et al., 2006) also resulted in a dose-dependent death of the PCs (Figure S2B), demonstrating that both signaling pathways downstream of stromal cell contacts and BCMA are nonredundant and essential for PC survival.

### Inhibition of PI3K Signaling Ablates Resident Memory PCs of the BM

To verify the relevance of PI3K signaling for the persistence of PC *in vivo*, we treated mice with an established immune memory with 1.2 mg/kg of Wortmannin (Nobs et al., 2015), 7 weeks following the last immunization, and enumerated memory PCs in the BM on day 95 (Figures 2D and 2E). Counts of total BM cells did not differ between mice treated with Wortmannin and control mice ($2.46 \times 10^8$ versus $2.37 \times 10^8$, respectively) (Figure 2F). However, PCs were significantly reduced by about 33% ($5.1 \times 10^5$ versus $3.4 \times 10^5$).These results show that the persistence of memory PCs in the BM, like in the *ex vivo* niche provided by ST2 cells and APRIL, is conditional on continued PI3K signaling.

### Stromal Cell Contact Downregulates the FoxO1/3 Pathway

PI3K activation leads to the downregulation of FoxO1 and FoxO3 (Haftmann et al., 2012; Huang et al., 2005; Plas and Thompson, 2003). BM PCs, when co-cultured with ST2 cells, significantly downregulated the expression of FoxO1 and FoxO3 independently of APRIL, already on day 1 of co-culture (FoxO1 geometric mean expression: APRIL: 1,820 ± 62, ST2: 1,374 ± 76, ST2+A: 1,348 ± 35; FoxO3 geometric mean expression: APRIL: 2,446 ± 282, ST2: 1,777 ± 134, ST2+A: 1,960 ± 106) (Figures 3A and 3B). Adding APRIL alone or in combination with ST2 cells did not affect the expression of FoxO1/3 proteins. To determine whether downregulation of FoxO1/3 expression is the critical event downstream of PI3K activation, FoxO1/3 expression was knocked down by using specific siRNA by 23% and 21%, respectively (Figures S2B and S2C). Knockdown of FoxO1/3 in PCs could completely restore PC survival in the absence of ST2 cells when PCs were cultured with APRIL alone (mean viability: day 1, APRIL+ scr: 99% ± 13%, APRIL+ FoxO: 102% ± 14%, ST2+A+scr: 101% ± 33%; day 3, APRIL+ scr: 52% ± 26%, APRIL+ FoxO: 79% ± 18%, ST2+A+scr: 89% ± 24%) (Figure 3C). These results demonstrate that stromal cell contact is supporting the survival of PCs by downregulation of FoxO1/3.

### Stromal Cell Contact and APRIL Signaling Pathways Address Distinct Caspases

As the survival of *ex-vivo*-isolated BM PCs cultured with APRIL or ST2 cells alone was rescued by pan-caspase inhibitors (Figure 1B), we aimed at determining which caspases are affected by stromal cell contact and APRIL, respectively. Activation of the caspases was measured on the single-cell level by using antibodies or fluorescent caspase-specific peptides and controlled using a pan-caspase inhibitor and Wortmannin, blocking PI3K signaling, or tunicamycin, inducing the unfolded protein response (UPR), respectively (Figure S3). Co-culture of PCs with ST2 cells led to significantly reduced levels of cleavage and activation of the effector Casp3 and 7 (Figures 4A and 4B) compared to levels of culture with APRIL alone. The addition of APRIL to the co-culture with ST2 cells did not further impact the activation of Casp3 or 7. However, APRIL, together with ST2 cells, led to a significant reduction of the activation of the ER-associated Casp12 (Figure 4C).

### Stromal Cell Contact and APRIL Synergize to Induce the Expression of IRF4

ST2 cells and APRIL also synergized to upregulate the expression of IRF4 on the single-cell level (Figure S2C). IRF4 has been demonstrated previously to be indispensable for the survival of PCs *in vivo* (Tellier et al., 2016). Inhibition of either

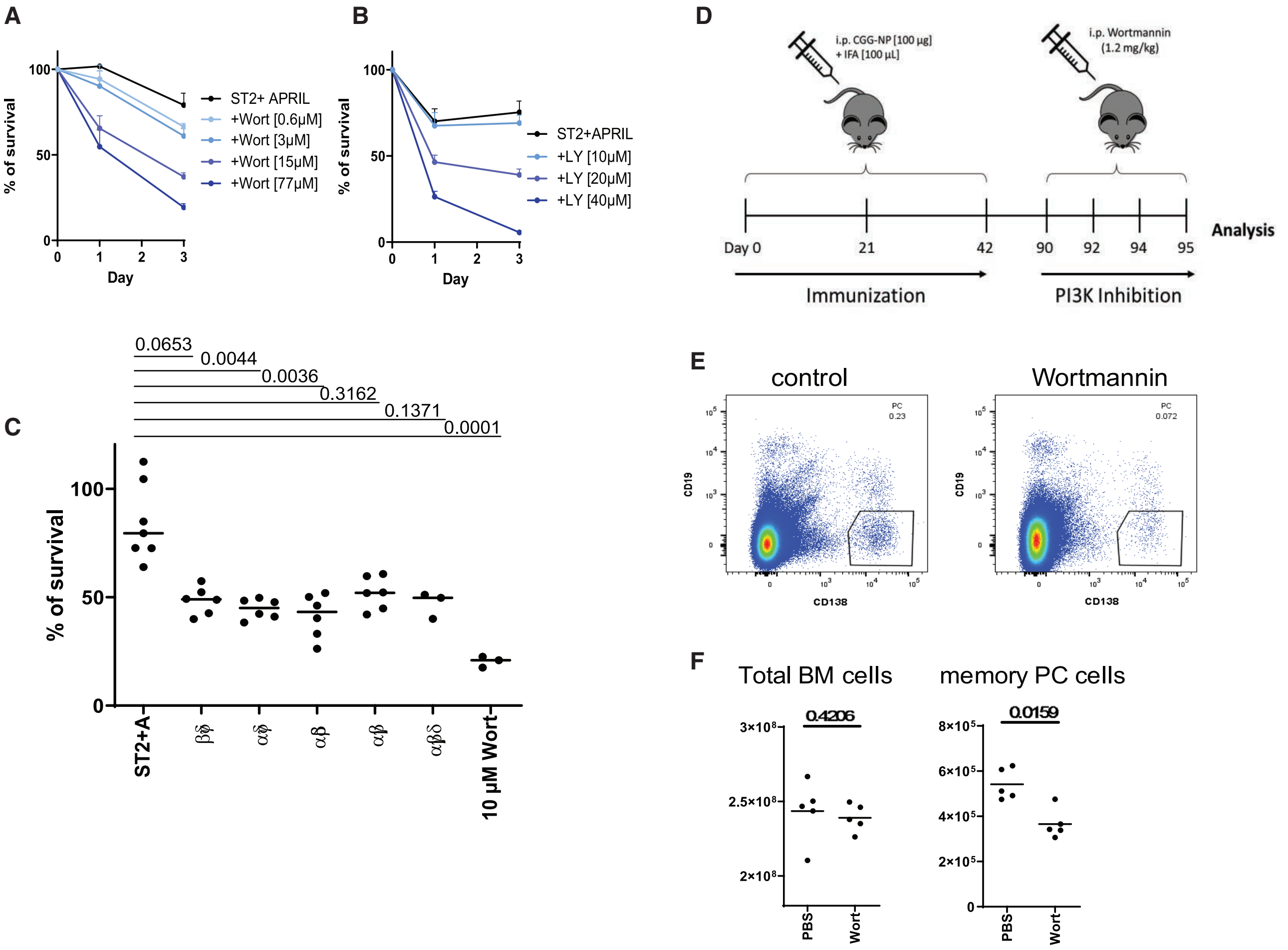


**Figure 2. Stromal-Cell-Contact-Induced PI3K Signaling Is Essential for Survival of Memory Bone Marrow PCs *Ex Vivo* and *In Vivo***

(A–C) Survival of PCs preincubated with different concentrations of the irreversible PI3K-inhibitor Wortmannin (A), directly treated during culture with the inhibitor LY294002 (B), or with combinations of three subunit-specific PI3K inhibitors determined by counting viable $CD138^{++}/DAPI^{-}$ PCs by flow cytometry (C) (pooled from two independent experiments with n = 6 technical replicates for each group). Statistics: ordinary one-way ANOVA.

(D) Experimental design: C57BL/6J were primed and boosted twice (days 21 and 42) with CGG-NP/IFA and treated with the PI3K-inhibitor Wortmannin on days 90, 92, and 94. On day 95, the mice were analyzed.

(E) Representative plot of $B220^{-}/CD138^{++}/CD19^{-}$ PCs in the bone marrow from control and Wortmannin-treated mice gated on $DAPI^{-}$ viable cells.

(F) Absolute cell counts of total bone marrow cells and memory PCs, in the bone marrow in control and Wortmannin-treated mice. Median of two pooled independent experiments with n = 5 biological replicates. Statistics: t test.

signaling pathways downstream of stromal cell contact or APRIL, i.e., PI3K using Wortmannin or NF-κB using IKK16, resulted in a decrease in IRF4 expression (Figure S2D).

## DISCUSSION

Long-lived memory PCs persist for a lifetime in mice (Manz et al., 1997; Slifka et al., 1998), in nonhuman primates (Hammarlund et al., 2017), and in humans (Landsverk et al., 2017). They form an independent population of memory cells (Chang et al., 2018; Radbruch et al., 2006). Memory PCs are evenly distributed throughout the BM and most if not all of them directly contact a mesenchymal stromal cell (Zehentmeier et al., 2014). It is suggestive that these stromal cells organize a survival niche, which supports longevity of the PCs (Manz and Radbruch, 2002). It is still enigmatic, however, how stromal cells organize the PC survival niche. It has been postulated that they attract cells secreting the cytokines APRIL and/or BAFF, both ligands for the receptor BCMA (CD269), signaling by the NF-κB pathway (Chu et al., 2011; Hatzoglou et al., 2000; O'Connor et al., 2004). Blocking both cytokines ablates PCs from the BM (Belnoue et al., 2008; Benson et al., 2008). The stromal cell may also contribute directly to the survival of PCs by integrin-mediated cell contact signaling (DiLillo et al., 2008) inducing PI3K signaling (Van Spriel et al., 2012). Here, we show that indeed both signaling pathways are essential and complementary for PC survival and that cell-contact-induced PI3K signaling acts through downregulation of FoxO1/3 and blocks activation of caspases 3 and 7, but not 12. In complementation, APRIL signaling blocks activation of Casp12, but not 3 and 7. Direct

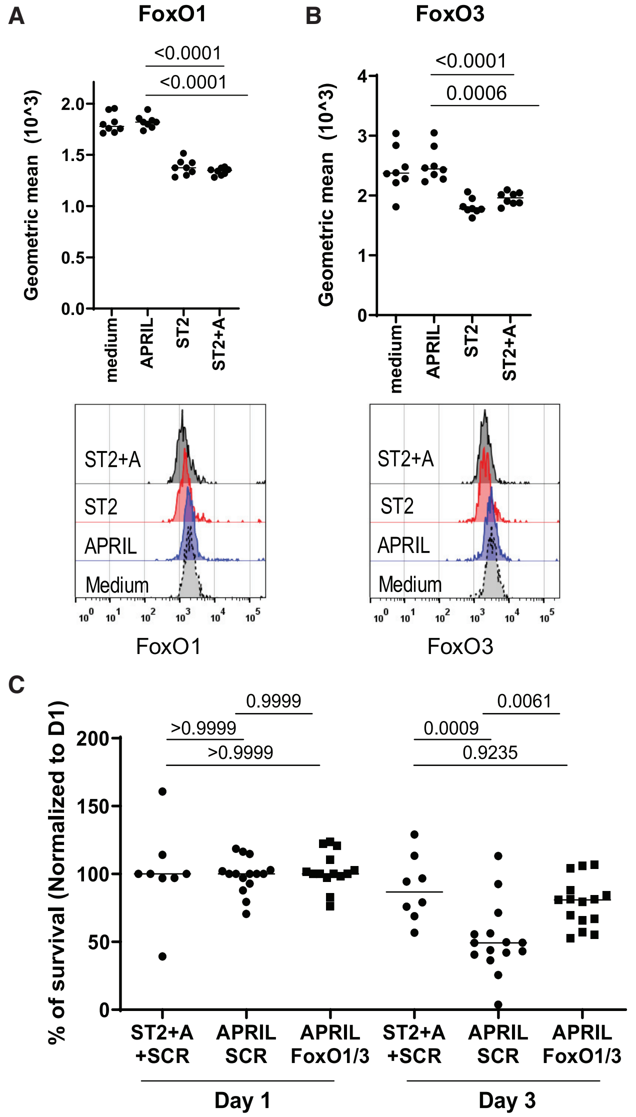


**Figure 3. Stromal-Cell-Contact-Induced PI3K Signaling Down-regulates FoxO1/3 Protein Expression and Is Essential for Survival of Memory Bone Marrow PCs**

(A and B) FoxO1 (A) and FoxO3 (B) expression of CD138$^{+}$ PCs, as determined by flow cytometry (geometric mean fluorescence intensity, pooled from two independent experiments with n = 8 technical replicates for each group). Statistics: ordinary one-way ANOVA.

(C) Survival of PCs treated with siRNA specific for FoxO1/FoxO3 or scrambled control on day 1 and 3 of culture with APRIL alone or ST2 and APRIL counted by flow cytometry. Median of at least three pooled independent experiments with n = 3 technical replicates for each group. Statistics: ordinary one-way ANOVA.

contact between the stromal cell and the PCs and APRIL signaling thus in synergy prevent apoptosis of the PCs induced by ER and mitochondrial stress. It has been shown previously that primary human-antibody-secreting cells isolated from peripheral blood 7 days after booster vaccination can be maintained *in vitro* by soluble factors secreted by BM-derived stromal cells (Nguyen et al., 2018), independently of direct cell-cell contact. It is unclear whether this apparent discrepancy to the results shown here points to a difference in survival requirements of antibody-secreting cells circulating in the blood, within days after antigenic stimulation, i.e., recently generated plasmablasts and long-lived PCs residing in the BM.

An analysis of the lifestyle of memory PCs has been hampered by their low frequency in the BM, as well as by the fact that they die rapidly when isolated and cultured *ex vivo* (Cassese et al., 2003), as is confirmed here. Cytokines prolonging their survival *ex vivo* have been reported (Cassese et al., 2003; Cocco et al., 2012; Jourdan et al., 2014; Minges Wols et al., 2002). Here, we describe a culture system providing stroma cell contact and a ligand for BCMA and show that those two signals are necessary and sufficient to maintain survival of resident PCs of the BM. When cultured on ST2 cells, in the presence of the cytokine APRIL, 50 to 80% of *ex-vivo*-isolated BM PCs survived for more than 5 days. The PCs maintained their transcriptional identity, with less than 41 transcripts differentially expressed between PCs directly isolated from the BM and those cultured for 3 days.

*In vivo*, it remains unclear which cells provide the cytokines APRIL and/or BAFF to the PCs. Eosinophilic granulocytes have been proposed (Chu et al., 2011), but their role has been questioned (Haberland et al., 2018). We have shown recently that a subpopulation of BM stromal cells does produce BAFF (Addo et al., 2019). PCs are known to have two receptors for the cytokines APRIL and BAFF, namely TACI and BCMA (O'Connor et al., 2004; Peperzak et al., 2013). Among them, BCMA signaling has been suggested to be vital for PC survival in the BM (Benson et al., 2008; O'Connor et al., 2004). It is known that the BCMA receptor, when activated by APRIL, signals by the TRAF/NF-κB/p38 pathway (Hatzoglou et al., 2000). Similarly, inhibition of the NF-κB pathway blocks survival of PCs in the *ex vivo* niche described here. In the presence of stromal cells, APRIL significantly inhibits the activation of Casp12, but not of Casp3 and 7. Casp12 is localized at the ER and is activated by ER stress, e.g., by an insufficient UPR (Nakagawa et al., 2000). It was previously shown that managing the UPR is essential for PC survival (Iwakoshi et al., 2003; Pelletier et al., 2006) because they are cells producing several thousand antibody molecules per second (Hibi & Dosch, 1986). Which ER stress pathway is relevant for PCs is still not clear. The genetic ablation of the three main ER stress pathways XBP-1 (Taubenheim et al., 2012), ATF6 (Aragon et al., 2012), and PERK (Lam et al., 2018) individually did not result in an ablation of antibody-secreting cells, indicating that there might be redundancy in the ER stress pathways in PCs, which are apparently all addressed by BCMA signaling.

The original notion that direct cell contact between stromal cells and PCs is essential for the survival of PCs in the BM comes from their ablation *in vivo* by antibodies against integrin α4β1 (VLA-4), a ligand of the stromal cell receptor VCAM1, and integrin αLβ2 (LFA-1), a ligand of the stromal cell receptor ICAM-1, which are both expressed by BM PCs (DiLillo et al., 2008). At a molecular level, it has been demonstrated that integrins can signal by the FAK/PI3K pathway (Giancotti and Ruoslahti, 1999). An indication that this may also happen in PCs comes from mice deficient for the tetraspanin CD37. In these mice, antibody-secreting cells showed impaired clustering of VLA4 and diminished PI3K

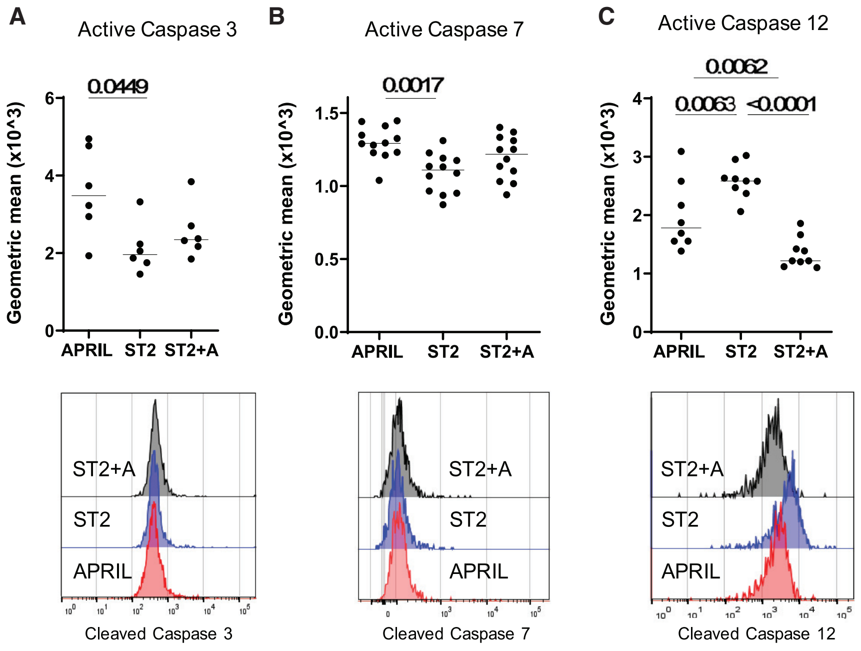


**Figure 4. Contact with Stromal Cells Inhibits Activation of Caspase 3 and 7, whereas APRIL Inhibits Activation of Caspase 12**

Expression of activated caspase 3 (A), caspase 7 (B), and caspase 12 (C) of CD138$^+$ PCs determined by flow cytometry, shown as geometric mean fluorescence intensity, on day 1 in PCs cultured under the indicated conditions (pooled from a minimum of two independent experiments with n = 6–12 technical replicates for each group). Statistics: Kruskal-Wallis test (activated Casp3), ordinary one-way ANOVA (activated Casp7/Casp12).

signaling *ex vivo* (Van Spriel et al., 2012). Here, we demonstrate by transwell separation of PCs and stromal cells in the *ex vivo* niche that direct contact of BM PCs to ST2 cells is required for the survival of the PCs. The contact induces PI3K signaling in the PCs, which is required for their survival. Inhibitors specific for the α, β, γ, or δ subunits of PI3K in any combination of three blocked PC survival, suggesting that PCs can use any two of the four different PI3K subunits for survival signaling in a redundant fashion. Also, the PI3K inhibitors LY29400 and Wortmannin efficiently block *ex vivo* survival of the PCs. Wortmannin also ablates PCs from BM *in vivo*. Considering the different spectra of off-target effects of all those inhibitors, the results strongly suggest that indeed stromal-cell-contact-induced PI3K signaling is vital for memory PC survival. The cell contact in part is mediated by β1-integrin because siRNA-mediated knockdown of β1 integrin in PCs impairs the survival of these cells in the *ex vivo* niche, as we show here.

PI3K signaling by activation of AKT has several downstream targets. One of them is the mechanistic target of rapamycin complex 1 (mTORC1). This has previously been shown not to be involved in memory PC survival (Jones et al., 2016). Others are the transcription factors FoxO1/3. Activated AKT phosphorylates FoxO1/3, thus inactivating them as transcription factors. Inactivation of FoxO1/3 has been shown to be essential for survival and proliferation of activated lymphocytes, although the exact molecular mechanisms remain enigmatic (Haftmann et al., 2012; Stittrich et al., 2010). Here, we show that inactivation of FoxO1/3 is essential for the survival of memory PCs because siRNA-mediated knockdown of FoxO1/3 in the PCs nearly fully compensates for stromal cell contact in the *ex vivo* niche.

Unlike APRIL, stromal cells block activation of the Casp3 and 7, but not 12, in BM PCs in the *ex vivo* niche. Casp3 and 7 have been reported to be activated upon mitochondrial stress (Lakhani et al., 2006). Inhibition of Casp3 and 7 by stromal cell contact and of Casp12 by APRIL seem to be necessary and sufficient to prevent apoptosis of memory PCs in the *ex vivo* niche. Pan-caspase inhibition can compensate for either one, ST2 cells or APRIL. Although stromal cell contact blocks the effector caspases of mitochondrial-stress-induced apoptosis, APRIL blocks the initiator caspase of ER-stress-induced apoptosis Casp12, which are apparently the two major stress factors limiting the lifetime of memory PCs. Also, the expression of the essential survival factor IRF4 (Tellier et al., 2016) is upregulated by stromal cell contact and APRIL in synergy, as we show here. Taken together, our results demonstrate that stromal cells are not simply organizing memory PC survival niches in the BM. By integrin-mediated cell contact, they actively provide an essential survival signal to memory PCs, complementing the survival signal provided by APRIL/BAFF. Both signals efficiently prevent mitochondrial- and ER-stress-induced apoptosis of memory PCs.

## STAR★METHODS

Detailed methods are provided in the online version of this paper and include the following:

- KEY RESOURCES TABLE
- RESOURCE AVAILABILITY
  - Lead Contact
  - Materials Availability
  - Data and Code Availability
- EXPERIMENTAL MODEL AND SUBJECT DETAILS
  - Mice
  - Health/immune status
  - Husbandry/housing conditions of experimental animals
  - ST2 cell line
- METHOD DETAILS
  - Immunization
  - *In vivo* treatment with PI3K-inhibitor
  - Magnetic isolation of long-lived PCs from the bone marrow
  - Cell culture of long-lived PCs and treatment with inhibitors

 - ○ siRNA treatment of PCs *in vitro*
 - ○ Caspase stainings
 - ○ Flow cytometric measurement of surface and intracellular antigens
 - ○ ELISA
 - ○ Transwell-Assay
 - ○ Processing and analysis of oligonucleotide microarray data
- ● QUANTIFICATION AND STATISTICAL ANALYSIS

## SUPPLEMENTAL INFORMATION

Supplemental Information can be found online at https://doi.org/10.1016/j.celrep.2020.107982.

## ACKNOWLEDGMENTS

We thank Tuula Geske, Heidi Hecker-Kia, and Heidi Schliemann for their expert technical help; Toralf Kaiser and Jenny Kirsch as operators of the flow cytometry core facility (FCCF); and Patrick Thiemann and Manuela Ohde for assistance with animal care. This work was supported by European Research Council Advanced Grant IMMEMO (ERC-2010-AdG.20100317 Grant 268987; to A.R.), by the Deutsche Forschungsgemeinschaft (TRR130 to A.R. and H.-D.C.), and Innovative Medicines Initiative 2 Joint Undertaking under grant agreement no. 777357. G.P. and F.M. were supported by the state of Berlin and the “European Regional Development Fund” (ERDF 2014–2020, EFRE 1.8/11, Deutsches Rheuma-Forschungszentrum Berlin). F.S. was supported by Osteoimmune, a FP7 Marie Curie Initial Training Network (FP7-PEOPLE-2011-ITN-289150). E.M. was supported by the Deutsche Forschungsgemeinschaft (MO 2934/1-1-1). This work was supported by the Leibniz ScienceCampus Chronic Inflammation (http://www.drfz.de/forschung/chronischen-entzuendungen).

## AUTHOR CONTRIBUTIONS

R.C. and S.H. designed and performed the experiments, analyzed and interpreted the data, and wrote the manuscript. D.M., M.T., C.K., L.H., and F.S. carried out and analyzed certain experiments. B.F.H. and F.H. provided mouse strains and helped to write the manuscript. G.P., A.T., P.D., E.M., K.T., F.M., and M.-F.M. analyzed data and provided scientific suggestions. H.-D.C. and A.R. conceived the study and wrote the manuscript.

## DECLARATION OF INTEREST

The authors declare no competing interests.

Received: December 12, 2019
Revised: May 25, 2020
Accepted: July 9, 2020
Published: August 4, 2020

## REFERENCES

Addo, R.K., Heinrich, F., Heinz, G.A., Schulz, D., Sercan-Alp, Ö., Lehmann, K., Tran, C.L., Bardua, M., Matz, M., Löhning, M., et al. (2019). Single-cell transcriptomes of murine bone marrow stromal cells reveal niche-associated heterogeneity. Eur. J. Immunol. *49*, 1372–1379.

Aragon, I.V., Barrington, R.A., Jackowski, S., Mori, K., and Brewer, J.W. (2012). The specialized unfolded protein response of B lymphocytes: ATF6α-independent development of antibody-secreting B cells. Mol. Immunol. *51*, 347–355.

Bardua, M., Haftmann, C., Durek, P., Westendorf, K., Buttgereit, A., Tran, C.L., McGrath, M., Weber, M., Lehmann, K., Addo, R.K., et al. (2018). MicroRNA-31 Reduces the Motility of Proinflammatory T Helper 1 Lymphocytes. Front. Immunol. *9*, 2813.

Belnoue, E., Pihlgren, M., McGaha, T.L., Tougne, C., Rochat, A.F., Bossen, C., Schneider, P., Huard, B., Lambert, P.H., and Siegrist, C.A. (2008). APRIL is critical for plasmablast survival in the bone marrow and poorly expressed by early-life bone marrow stromal cells. Blood *111*, 2755–2764.

Benson, M.J., Dillon, S.R., Castigli, E., Geha, R.S., Xu, S., Lam, K.-P., and Noelle, R.J. (2008). Cutting edge: the dependence of plasma cells and independence of memory B cells on BAFF and APRIL. J. Immunol. *180*, 3655–3659.

Cassese, G., Arce, S., Hauser, A.E., Lehnert, K., Moewes, B., Mostarac, M., Muehlinghaus, G., Szyska, M., Radbruch, A., and Manz, R.A. (2003). Plasma cell survival is mediated by synergistic effects of cytokines and adhesion-dependent signals. J. Immunol. *171*, 1684–1690.

Chang, H.D., Tokoyoda, K., and Radbruch, A. (2018). Immunological memories of the bone marrow. Immunol. Rev. *283*, 86–98.

Chu, V.T., Fröhlich, A., Steinhauser, G., Scheel, T., Roch, T., Fillatreau, S., Lee, J.J., Löhning, M., and Berek, C. (2011). Eosinophils are required for the maintenance of plasma cells in the bone marrow. Nat. Immunol. *12*, 151–159.

Cocco, M., Stephenson, S., Care, M.A., Newton, D., Barnes, N.A., Davison, A., Rawstron, A., Westhead, D.R., Doody, G.M., and Tooze, R.M. (2012). In vitro generation of long-lived human plasma cells. J. Immunol. *189*, 5773–5785.

Cossarizza, A., Chang, H.-D., Radbruch, A., Akdis, M., Andrä, I., Annunziato, F., Bacher, P., Barnaba, V., Battistini, L., Bauer, W.M., et al. (2017). Guidelines for the use of flow cytometry and cell sorting in immunological studies. Eur. J. Immunol. *47*, 1584–1797.

DiLillo, D.J., Hamaguchi, Y., Ueda, Y., Yang, K., Uchida, J., Haas, K.M., Kelsoe, G., and Tedder, T.F. (2008). Maintenance of long-lived plasma cells and serological memory despite mature and memory B cell depletion during CD20 immunotherapy in mice. J. Immunol. *180*, 361–371.

Gautier, L., Cope, L., Bolstad, B.M., and Irizarry, R.A. (2004). affy—analysis of Affymetrix GeneChip data at the probe level. Bioinformatics *20*, 307–315.

Giancotti, F.G., and Ruoslahti, E. (1999). Integrin signaling. Science *285*, 1028–1032.

Haberland, K., Ackermann, J.A., Ipseiz, N., Culemann, S., Pracht, K., Englbrecht, M., Jäck, H.-M., Schett, G., Schuh, W., and Krönke, G. (2018). Eosinophils are not essential for maintenance of murine plasma cells in the bone marrow. Eur. J. Immunol. *48*, 822–828.

Haftmann, C., Stittrich, A.B., Sgouroudis, E., Matz, M., Chang, H.D., Radbruch, A., and Mashreghi, M.F. (2012). Lymphocyte signaling: regulation of FoxO transcription factors by microRNAs. Ann. N Y Acad. Sci. *1247*, 46–55.

Haftmann, C., Riedel, R., Porstner, M., Wittmann, J., Chang, H.D., Radbruch, A., and Mashreghi, M.F. (2015). Direct uptake of Antagomirs and efficient knockdown of miRNA in primary B and T lymphocytes. J. Immunol. Methods *426*, 128–133.

Hammarlund, E., Thomas, A., Amanna, I.J., Holden, L.A., Slayden, O.D., Park, B., Gao, L., and Slifka, M.K. (2017). Plasma cell survival in the absence of B cell memory. Nat. Commun. *8*, 1781.

Hatzoglou, A., Roussel, J., Bourgeade, M.F., Rogier, E., Madry, C., Inoue, J., Devergne, O., and Tsapis, A. (2000). TNF receptor family member BCMA (B cell maturation) associates with TNF receptor-associated factor (TRAF) 1, TRAF2, and TRAF3 and activates NF-kappa B, elk-1, c-Jun N-terminal kinase, and p38 mitogen-activated protein kinase. J. Immunol. *165*, 1322–1330.

Hibi, T., and Dosch, H.-M. (1986). Limiting dilution analysis of the B cell compartment in human bone marrow. Eur. J. Immunol. *16*, 139–145.

Huang, H., Regan, K.M., Wang, F., Wang, D., Smith, D.I., van Deursen, J.M.A., and Tindall, D.J. (2005). Skp2 inhibits FOXO1 in tumor suppression through ubiquitin-mediated degradation. Proc. Natl. Acad. Sci. USA *102*, 1649–1654.

Iwakoshi, N.N., Lee, A.H., and Glimcher, L.H. (2003). The X-box binding protein-1 transcription factor is required for plasma cell differentiation and the unfolded protein response. Immunol. Rev. *194*, 29–38.

Jones, D.D., Gaudette, B.T., Wilmore, J.R., Chernova, I., Bortnick, A., Weiss, B.M., and Allman, D. (2016). mTOR has distinct functions in generating versus sustaining humoral immunity. J. Clin. Invest. *126*, 4250–4261.

Jourdan, M., Cren, M., Robert, N., Bolloré, K., Fest, T., Duperray, C., Guilloton, F., Hose, D., Tarte, K., and Klein, B. (2014). IL-6 supports the generation of human long-lived plasma cells in combination with either APRIL or stromal cell-soluble factors. Leukemia *28*, 1647–1656.

Lakhani, S.A., Masud, A., Kuida, K., Porter, G.A., Jr., Booth, C.J., Mehal, W.Z., Inayat, I., and Flavell, R.A. (2006). Caspases 3 and 7: key mediators of mitochondrial events of apoptosis. Science *311*, 847–851.

Lam, W.Y., Jash, A., Yao, C.H., D'Souza, L., Wong, R., Nunley, R.M., Meares, G.P., Patti, G.J., and Bhattacharya, D. (2018). Metabolic and Transcriptional Modules Independently Diversify Plasma Cell Lifespan and Function. Cell Rep. *24*, 2479–2492.e6.

Landsverk, O.J.B., Snir, O., Casado, R.B., Richter, L., Mold, J.E., Réu, P., Horneland, R., Paulsen, V., Yaqub, S., Aandahl, E.M., et al. (2017). Antibody-secreting plasma cells persist for decades in human intestine. J. Exp. Med. *214*, 309–317.

Makela, O., and Nossal, G.J. (1962). Autoradiographic studies on the immune response. II. DNA synthesis amongst single antibody-producing cells. J. Exp. Med. *115*, 231–244.

Manz, R.A., and Radbruch, A. (2002). Plasma cells for a lifetime? Eur. J. Immunol. *32*, 923–927.

Manz, R.A., Thiel, A., and Radbruch, A. (1997). Lifetime of plasma cells in the bone marrow. Nature *388*, 133–134.

Minges Wols, H.A., Underhill, G.H., Kansas, G.S., and Witte, P.L. (2002). The role of bone marrow-derived stromal cells in the maintenance of plasma cell longevity. J. Immunol. *169*, 4213–4221.

Nakagawa, T., Zhu, H., Morishima, N., Li, E., Xu, J., Yankner, B.A., and Yuan, J. (2000). Caspase-12 mediates endoplasmic-reticulum-specific apoptosis and cytotoxicity by amyloid-beta. Nature *403*, 98–103.

Nguyen, D.C., Garimalla, S., Xiao, H., Kyu, S., Albizua, I., Galipeau, J., Chaing, K.-Y., Waller, E.K., Wu, R., Gibson, G., et al. (2018). Factors of the bone marrow microniche that support human PC survival and immunoglobulin secretion. Nat. Commun. *9*, 3698.

Nobs, S.P., Schneider, C., Dietrich, M.G., Brocker, T., Rolink, A., Hirsch, E., and Kopf, M. (2015). PI3-Kinase-$\gamma$ Has a Distinct and Essential Role in Lung-Specific Dendritic Cell Development. Immunity *43*, 674–689.

O'Connor, B.P., Raman, V.S., Erickson, L.D., Cook, W.J., Weaver, L.K., Ahonen, C., Lin, L.-L., Mantchev, G.T., Bram, R.J., and Noelle, R.J. (2004). BCMA is essential for the survival of long-lived bone marrow plasma cells. J. Exp. Med. *199*, 91–98.

Parsons, J.T., Martin, K.H., Slack, J.K., Taylor, J.M., and Weed, S.A. (2000). Focal adhesion kinase: a regulator of focal adhesion dynamics and cell movement. Oncogene *19*, 5606–5613.

Pelletier, N., Casamayor-Pallejà, M., De Luca, K., Mondière, P., Saltel, F., Jurdic, P., Bella, C., Genestier, L., and Defrance, T. (2006). The endoplasmic reticulum is a key component of the plasma cell death pathway. J. Immunol. *176*, 1340–1347.

Peperzak, V., Vikström, I., Walker, J., Glaser, S.P., LePage, M., Coquery, C.M., Erickson, L.D., Fairfax, K., Mackay, F., Strasser, A., et al. (2013). Mcl-1 is essential for the survival of plasma cells. Nat. Immunol. *14*, 290–297.

Plas, D.R., and Thompson, C.B. (2003). Akt activation promotes degradation of tuberin and FOXO3a via the proteasome. J. Biol. Chem. *278*, 12361–12366.

Radbruch, A., Muehlinghaus, G., Luger, E.O., Inamine, A., Smith, K.G.C., Dörner, T., and Hiepe, F. (2006). Competence and competition: the challenge of becoming a long-lived plasma cell. Nat. Rev. Immunol. *6*, 741–750.

Ritchie, M.E., Phipson, B., Wu, D., Hu, Y., Law, C.W., Shi, W., and Smyth, G.K. (2015). limma powers differential expression analyses for RNA-sequencing and microarray studies. Nucleic Acids Res. *43*, e47.

Schooley, J.C. (1961). Autoradiographic observations of plasma cell formation. J. Immunol. *86*, 331–337.

Slifka, M.K., Antia, R., Whitmire, J.K., and Ahmed, R. (1998). Humoral immunity due to long-lived plasma cells. Immunity *8*, 363–372.

Spencer, J.A., Ferraro, F., Roussakis, E., Klein, A., Wu, J., Runnels, J.M., Zaher, W., Mortensen, L.J., Alt, C., Turcotte, R., et al. (2014). Direct measurement of local oxygen concentration in the bone marrow of live animals. Nature *508*, 269–273.

Stittrich, A.B., Haftmann, C., Sgouroudis, E., Kühl, A.A., Hegazy, A.N., Panse, I., Riedel, R., Flossdorf, M., Dong, J., Fuhrmann, F., et al. (2010). The microRNA miR-182 is induced by IL-2 and promotes clonal expansion of activated helper T lymphocytes. Nat. Immunol. *11*, 1057–1062.

Taubenheim, N., Tarlinton, D.M., Crawford, S., Corcoran, L.M., Hodgkin, P.D., and Nutt, S.L. (2012). High rate of antibody secretion is not integral to plasma cell differentiation as revealed by XBP-1 deficiency. J. Immunol. *189*, 3328–3338.

Tellier, J., Shi, W., Minnich, M., Liao, Y., Crawford, S., Smyth, G.K., Kallies, A., Busslinger, M., and Nutt, S.L. (2016). Blimp-1 controls plasma cell function through the regulation of immunoglobulin secretion and the unfolded protein response. Nat. Immunol. *17*, 323–330.

Thein, S., Pham, A., Bayer, K.U., Tao-Cheng, J.-H., and Dosemeci, A. (2014). IKK Regulates the Deubiquitinase CYLD at the Postsynaptic Density. Biochem Biophys. Res. Commun. *450*, 550–554.

Tokoyoda, K., Egawa, T., Sugiyama, T., Choi, B.-I., and Nagasawa, T. (2004). Cellular niches controlling B lymphocyte behavior within bone marrow during development. Immunity *20*, 707–718.

Van Spriel, A.B., De Keijzer, S., Van Der Schaaf, A., Gartlan, K.H., Sofi, M., Light, A., Linssen, P.C., Boezeman, J.B., Zuidscherwoude, M., Reinieren-Beeren, I., et al. (2012). The Tetraspanin CD37 Orchestrates the a 4 b 1 Integrin – Akt Signaling Axis and Supports Long-Lived PC Survival. Sci. Signal. *5*, ra82.

Waelchli, R., Bollbuck, B., Bruns, C., Buhl, T., Eder, J., Feifel, R., Hersperger, R., Janser, P., Revesz, L., Zerwes, H.-G., and Schlapbach, A. (2006). Design and preparation of 2-benzamido-pyrimidines as inhibitors of IKK. Bioorg. Med. Chem. Lett. *16*, 108–112.

Zehentmeier, S., Roth, K., Cseresnyes, Z., Sercan, Ö., Horn, K., Niesner, R.A., Chang, H.-D., Radbruch, A., and Hauser, A.E. (2014). Static and dynamic components synergize to form a stable survival niche for bone marrow plasma cells. Eur. J. Immunol. *44*, 2306–2317.

CellPress
OPEN ACCESS

## STAR★METHODS

### KEY RESOURCES TABLE

| REAGENT or RESOURCE | SOURCE | IDENTIFIER |
|---|---|---|
| Antibodies | | |
| Anti-mouse active caspase 3 | Cell Signaling Technology | Catalog # 8788; RRID:AB_2797665 |
| Anti-mouse active caspase 7 | Cell Signaling Technology | Catalog # 8438T; RRID:AB_11178377 |
| Anti-mouse BCL2, APC, REA356 | Miltenyi Biotec | Catalog # 130-105-474; RRID:AB_2651266 |
| Anti-mouse BIM, 14A8 | Milipore | Catalog # MAB17001; RRID:AB_2065314 |
| Anti-mouse CD138, PE-vio770, REA104 | Miltenyi Biotec | Catalog # 130-102-318; RRID:AB_2655025 |
| Anti-mouse FoxO1, C29H4 | Cell Signaling Technology | Catalog # 2880; RRID:AB_2106495 |
| Anti-mouse FoxO3a, D19A7 | Cell Signaling Technology | Catalog # 12829; RRID:AB_2636990 |
| Anti-mouse IRF4, APC, REA201 | Miltenyi Biotec | Catalog # 130-100-913; RRID:AB_2652517 |
| Anti-mouse MCL1, Y37 | Abcam | Catalog # ab32087; RRID:AB_776245 |
| Anti-mouse NOXA, 114C307 | Abcam | Catalog # ab13654; RRID:AB_300536 |
| Anti-mouse ki67,A488, B56 | BD | Catalog # 558616; RRID:AB_647087 |
| Anti-mouse B220, PE, REA755 | Miltenyi Biotec | Catalog # 130-110-709; RRID:AB_2658276 |
| Anti-mouse B220, Bio, RA3-6B2 | Miltenyi Biotec | Catalog # 130-101-928; RRID:AB_2660454 |
| Anti-mouse CD19, APC, REA749 | Miltenyi Biotec | Catalog # 130-112-036; RRID:AB_2655824 |
| Anti-mouse CD49d, bio, R1-2 | Miltenyi Biotec | Catalog # 130-101-912; RRID:AB_2660744 |
| Anti-mouse IgA, polyclonal | Southern Biotech | Catalog # 1040-08; RRID:AB_2794374 |
| Anti-mouse IgG, polyclonal | Southern Biotech | Catalog # 1036-01; RRID:AB_2794345 |
| Anti-mouse IgM, polyclonal | Southern Biotech | Catalog # 1021-01; RRID:AB_2687524 |
| Anti-mouse CD19, PacB, 1D3 | DRFZ | N/A |
| Chemicals, Peptides, and Recombinant Proteins | | |
| 4-hydroxy-3-nitropheylacetyl hapten coupled chicken gamma globulin | Biomol | Catalog # D602-0100 |
| Incomplete Freud's Adjuvans | SIGMA | Catalog # F5506 |
| Wortmannin | Selleckchem | Catalog # S2758 |
| Ly294002 | Selleckchem | Catalog # S1105 |
| IKK16 | Selleckchem | Catalog # S2882 |
| Z-VAD-FMK | Santa Cruz Biotechnology | Catalog# CAS 187389-52-2 |
| Formaldehyde solution | Electron microscopy | Catalog # 15713S |
| Recombinant APRIL mouse multimeric | Adipo gen Life Sciences | Catalog # AG-40B-0089-3010 |
| Anti-mouse CD138 microbeads | Miltenyi Biotech | Catalog # 130-098-257 |
| Anti-Streptavidin microbeads | Miltenyi Biotech | Catalog # 130-048-101 |
| Oligonucleotides | | |
| FoxO1 | SMARTPool | Catalog # E-041127-00-0010 |
| FoxO3 | SMARTPool | Catalog # E-040728-00-0010 |
| ITGB1 | Individual siRNA | Catalog # A-040783-13-0020 |
| Non-Targeting | Individual siRNA | Catalog # D-001910-04-20 |
| Critical Commercial Assays | | |
| RNeasy Micro KIT | Quiagen | Catalog # 74004 |
| Whole-transcriptome pico KIT | ThermoFisher Scientific | Catalog # 902622 |
| Cell Signaling Buffer Set A | Miltenyi Biotec | Catalog # 130-100-827 |
| CaspGLOW Fluorescein Active Caspase-12 Staining Kit | Biovision GmbH | Catalog # K172-100 |

(*Continued on next page*)

| *Continued* | | |
|---|---|---|
| REAGENT or RESOURCE | SOURCE | IDENTIFIER |
| Deposited Data | | |
| Transcriptome data | This paper | GEO: GSE107206 |
| Experimental Models: Cell Lines | | |
| ST2 stromal cell line | Riken BioResource Center | Catalog # RCB0224 |
| Experimental Models: Organisms/Strains | | |
| Mouse: C57BL/6J | Charles River Laboratories | Catalog # 000664 |
| Mouse: C57BL/6J Blimp-1:GFP | S. Nutt (Walter and Eliza Hall Institute, Melbourne, Australia) | N/A |
| Software and Algorithms | | |
| FlowJo10 | FlowJo LLC | http://www.flowjo.com |
| Prism | GraphPad Software, Inc | http://www.graphpad.com |
| Other | | |
| MG_U430_2 GeneChips | ThermoFisher Scientific | Catalog # 900495 |

## RESOURCE AVAILABILITY

### Lead Contact
Further information and requests for resources and reagents should be directed to and will be fulfilled by the Lead Contact, Andreas Radbruch (radbruch@drfz.de).

### Materials Availability
This study did not generate new unique reagents.

### Data and Code Availability
The datasets generated during this study are available at GEO: GSE107206.

## EXPERIMENTAL MODEL AND SUBJECT DETAILS

### Mice
C57BL/6J wild-type strain was purchased from Charles River Laboratories. Mice expressing GFP under the control of the Prdm1 promoter (Blimp-1:GFP) were bred and maintained at the "Bundesinstitut für Risikobewertung" (BfR, Berlin, Germany), a gift from S. Nutt (Walter and Eliza Hall Institute, Melbourne, Australia). All mice were maintained under specific pathogen free conditions. All experiments were performed according to German law for animal protection and with the permission from the local veterinary offices, and in compliance with the guidelines of the Institutional Animal Care and Use Committee. The animal experiments were performed within the animal studies H007015 and G008-13.

### Health/immune status
All animals are fully immune competent and are obtained from controlled SPF-breeding facilities. They are subsequently housed in IVC cages under SPF conditions. Animal health is constantly monitored in accordance with FELASA recommendations.

### Husbandry/housing conditions of experimental animals
All animals are housed in IVC cages with a maximum of 5 animals per cage. The animals are provided with enrichment in form of nesting material and wood. Special, autoclaved food as well as autoclaved water is supplied *ad libitum*. Cages are equipped with wood chip bedding material and a shelter made from red plastics. Animals have a settling-in-period of five days after arrival from breeding facilities. Animals are exposed to light for a cycle of 12 hours, followed by 12 hours of darkness.

### ST2 cell line
ST2 cells are originally isolated from the bone marrow of a BALB/c mouse. Their morphology is fibroblast-like. Cells were cultured in RPMI1640 + 10% FCS, 100 U/ml Penicillin, 100 μg/ml streptomycin, 0.1% β-Mercaptoethanol. Cells were passaged twice a week using trypsin, the passage ratio was 1:10.

## METHOD DETAILS

### Immunization

Mice were primed with 100μg 4-hydroxy-3-nitropheylacetyl hapten coupled chicken gamma globulin (NP-CGG) in incomplete Freud's Adjuvants (IFA) intraperitoneally (i.p.). In total 200μl (100μl of NP-CGG diluted in PBS + 100μl IFA) have been injected per mouse. Mice were challenged twice after the prime with the same injection in cycles of 21 days.

### *In vivo* treatment with PI3K-inhibitor

Immunized mice were used and Wortmannin in DMSO or PBS in DMSO injected i.p. with a total volume of 100μl on day 90, 92 and 94. Concentration of Wortmannin was 1.2mg/kg. Mice were killed by cervical dislocation and analyzed on day 95.

### Magnetic isolation of long-lived PCs from the bone marrow

PCs were magnetically isolated from immunized mice more than 30 days after $2^{nd}$ boost using a two-step protocol, including depletion of B220 and CD49b expressing cells and subsequent positive enrichment of $CD138^{high}$ PCs.

### Cell culture of long-lived PCs and treatment with inhibitors

Isolated long-lived PCs from the bone marrow were cultured in RPMI1640 medium supplemented with 10% FCS, 100 U/ml Penicillin, 100 μg/ml streptomycin, 0.1% β-Mercaptoethanol, 25 mM HEPES buffer and 50 ng/ml multimeric APRIL. Cultures were kept under physiological oxygen levels in a hypoxia chamber with 4.2% $O_2$ and 5% $CO_2$ at 37°C. For the co-culture, 2500 ST2 cells were seeded in a 96-well plate one day before memory PC isolation. Memory PCs were plated on top the ST2 cell layer in a 1:1 ratio (5000 PC and 5000 ST2 cells). For analysis, cells were either fixed with PFA or stained directly and scraped off the plate before measurement. Cells were pre-treated with pan PI3K inhibitors at different concentrations including Wortmannin, Ly294002 and to block NF-κB pathway the inhibitor, IKK16, was used.

### siRNA treatment of PCs *in vitro*

Isolated long-lived PCs from the bone marrow were cultured in Accell Medium with 2 μM siRNA or scr control (Bardua et al., 2018; Haftmann et al., 2015) and 100 ng/ml multimeric APRIL. After 1 hour RMI1640 medium supplemented with 5% FCS, 200 U/ml Penicillin, 200 μg/ml streptomycin0.2% β-Mercaptoethanol, 50 mM HEPES buffer was added to the cells. Cultures were kept under physiological oxygen levels in a hypoxia chamber with 4.2% $O_2$ and 5% $CO_2$ at 37°C.

### Caspase stainings

For activated caspase 12, Caspase 12 CaspGlow assay was performed by staining the cells in cell culture medium with the Casp-Glow FITC labeled peptide for 30 minutes at 37°C. Cells were washed two times, stained for CD138 and measured by flow cytometry. For caspases 3 and 7, specific antibodies were used. Cells were fixed with 4% PFA for 10 minutes and permeabilized with methanol. To block unspecific binding, cells were incubated with uncoupled anti-FCyRII/III antibody prior to staining with primary antibody for 1 hour. After washing, cells were incubated with a secondary antibody for 30 minutes. All samples were analyzed using a MacsQuant analyzer and FlowJo software. As negative control, cells were incubated for 24 hours with 10 μM panCaspase Inhibitor Z-VAD-FMK. As positive controls, cells were incubated with 10 μM Wortmannin or 2.5 μM Tunicamycin for 2 hours prior to staining.

### Flow cytometric measurement of surface and intracellular antigens

Single cell suspension was prepared; cells were pre-incubated with uncoupled anti-FCyRII/III antibody to block unspecific binding, followed by direct addition of antibodies for 15 minutes on ice. For staining of intracellular antigens, cells were fixed with PFA and permeabilized with methanol. To prevent unspecific binding, cells were pre-incubated with blocking buffer containing rabbit or rat serum and subsequently stained with primary antibody for 1 hour and, if necessary, with secondary antibody for 30 minutes. Samples were analyzed using a MacsQuant analyzer and FlowJo software. Cytometric procedures followed the recommendations of the "Guidelines for use of flow cytometry and cell sorting in immunological studies" (Cossarizza et al., 2017).

### ELISA

Enzyme-linked immunosorbent assay was used to detect secreted antibodies in supernatants at different time points of the PC culture. The supernatant was collected over 6 days. Plates were coated with unlabeled IgG, IgM or IgA in PBS overnight, followed by a washing step with PBS. Plates were blocked with 3% BSA/PBS. Supernatants either undiluted or diluted 1:12, 1:32 or 1:108 were incubated at 37°C for 2 hours in plates coated with unlabeled IgG, IgM or IgA. Plates were washed with PBS/Tween 0.05% followed by addition of anti-IgG, anti-IgM and anti-IgA conjugated to Biotin (diluted 1:2000) and incubation at 4°C overnight. Plates were washed with PBS/BSA and incubated with Streptavidin-Peroxidase at 37°C for 20 minutes. Plates were washed with tap water. TMB was added followed by stop solution (2M $H_2SO_4$). For the read out a spectrophotometer was used (wavelenght 450 nm).

### Transwell-Assay

Isolated PCs were cultured in transwell plates with pore size of 0.5μm. PCs were plated at the bottom of the transwell plate in the presence of APRIL in a total volume of 600 μL. 2500 stromal cells were plated the day before in the transwell insert. As control of direct contact effects PCs were plated directly onto stromal cells in the transwell insert. On the day of analysis, plasma cells were collected by scraping and directly measured using CD138 and DAPI staining.

### Processing and analysis of oligonucleotide microarray data

Memory PCs were isolated by magnetic cell sorting or after 3 days of culture in the *in vitro* system were processed for RNA preparation. RNA was prepared using the RNeasy Micro KIT and hybridized to mouse 430 2 GeneChips according to the whole-transcriptome pico KIT. Raw signals were processed by the affy R package using RMA for normalization (Gautier et al., 2004). Sample similarity was evaluated by Pearson correlation and Principle component analysis based on un-scaled log2 expression values. For the differentially expressed gene analysis the limma R package was used (Ritchie et al., 2015). Genes with an adjusted P value $< 0.05$ were considered to be statistically differentially expressed. Microarray data is available through Gene Expression Omnibus (GEO:GSE107206; code for reviewer: sfctqoakblqhpyp).

## QUANTIFICATION AND STATISTICAL ANALYSIS

Data are presented as median. Data was tested for normality and significant differences between two or more groups were determined by performing Mann-Whitney test, Kruskal-Wallis test or ordinary one-way ANOVA. Differences were considered statistically significant when $p < 0.05$. Detailed information can be found in the respective figure legend. Technical replicates refer to the number of wells in a 96 well plate of one experiment. Biological replicates refer to independent experiments performed on different days.

**Cell Reports, Volume *32***

## Supplemental Information

# Stromal Cell-Contact Dependent PI3K and APRIL Induced NF-κB Signaling Prevent Mitochondrial- and ER Stress Induced Death of Memory Plasma Cells

**Rebecca Cornelis, Stefanie Hahne, Adriano Taddeo, Georg Petkau, Darya Malko, Pawel Durek, Manja Thiem, Lukas Heiberger, Lena Peter, Elodie Mohr, Cora Klaeden, Koji Tokoyoda, Francesco Siracusa, Bimba Franziska Hoyer, Falk Hiepe, Mir-Farzin Mashreghi, Fritz Melchers, Hyun-Dong Chang, and Andreas Radbruch**

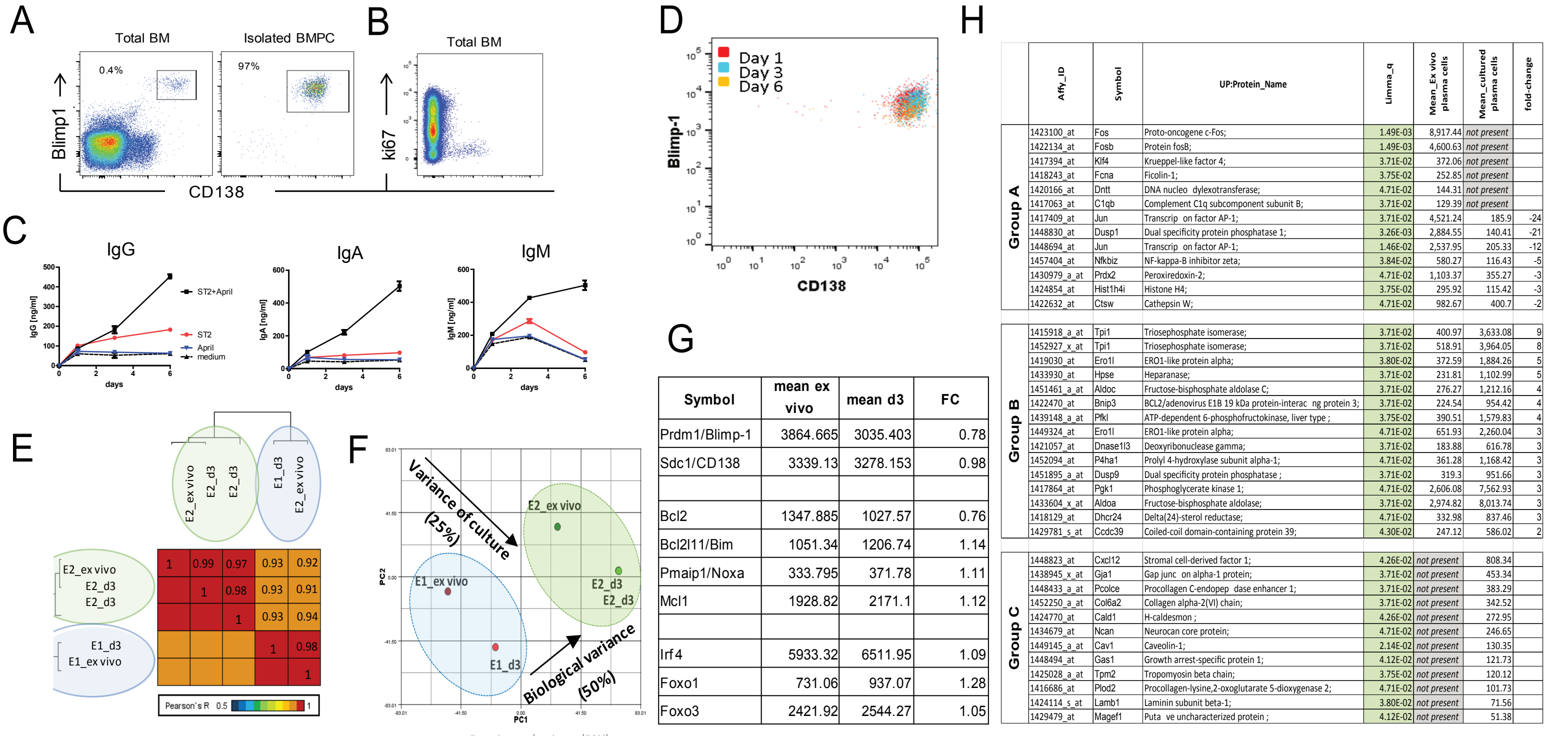


G

| Symbol | mean ex vivo | mean d3 | FC |
|---|---|---|---|
| Prdm1/Blimp-1 | 3864.665 | 3035.403 | 0.78 |
| Sdc1/CD138 | 3339.13 | 3278.153 | 0.98 |
| | | | |
| Bcl2 | 1347.885 | 1027.57 | 0.76 |
| Bcl2l11/Bim | 1051.34 | 1206.74 | 1.14 |
| Pmaip1/Noxa | 333.795 | 371.78 | 1.11 |
| Mcl1 | 1928.82 | 2171.1 | 1.12 |
| | | | |
| Irf4 | 5933.32 | 6511.95 | 1.09 |
| Foxo1 | 731.06 | 937.07 | 1.28 |
| Foxo3 | 2421.92 | 2544.27 | 1.05 |

H

| | Affy_ID | Symbol | UP:Protein_Name | Limma_q | Mean_Ex vivo plasma cells | Mean_cultured plasma cells | fold-change |
|---|---|---|---|---|---|---|---|
| Group A | 1423100_at | Fos | Proto-oncogene c-Fos; | 1.49E-03 | 8,917.44 | not present | |
| | 1422134_at | Fosb | Protein fosB; | 1.49E-03 | 4,600.63 | not present | |
| | 1417394_at | Klf4 | Krueppel-like factor 4; | 3.71E-02 | 372.06 | not present | |
| | 1418243_at | Fcna | Ficolin-1; | 3.75E-02 | 252.85 | not present | |
| | 1420166_at | Dntt | DNA nucleo dylexotransferase; | 4.71E-02 | 144.31 | not present | |
| | 1417063_at | C1qb | Complement C1q subcomponent subunit B; | 3.71E-02 | 129.39 | not present | |
| | 1417409_at | Jun | Transcrip on factor AP-1; | 3.71E-02 | 4,521.24 | 185.9 | -24 |
| | 1448830_at | Dusp1 | Dual specificity protein phosphatase 1; | 3.26E-03 | 2,884.55 | 140.41 | -21 |
| | 1448694_at | Jun | Transcrip on factor AP-1; | 1.46E-02 | 2,537.95 | 205.33 | -12 |
| | 1457404_at | Nfkbiz | NF-kappa-B inhibitor zeta; | 3.84E-02 | 580.27 | 116.43 | -5 |
| | 1430979_a_at | Prdx2 | Peroxiredoxin-2; | 4.71E-02 | 1,103.37 | 355.27 | -3 |
| | 1424854_at | Hist1h4i | Histone H4; | 3.75E-02 | 295.92 | 115.42 | -3 |
| | 1422632_at | Ctsw | Cathepsin W; | 4.71E-02 | 982.67 | 400.7 | -2 |
| Group B | 1415918_a_at | Tpi1 | Triosephosphate isomerase; | 3.71E-02 | 400.97 | 3,633.08 | 9 |
| | 1452927_x_at | Tpi1 | Triosephosphate isomerase; | 3.71E-02 | 518.91 | 3,964.05 | 8 |
| | 1419030_at | Ero1l | ERO1-like protein alpha; | 3.80E-02 | 372.59 | 1,884.26 | 5 |
| | 1433930_at | Hpse | Heparanase; | 3.71E-02 | 231.81 | 1,102.99 | 5 |
| | 1451461_a_at | Aldoc | Fructose-bisphosphate aldolase C; | 3.71E-02 | 276.27 | 1,212.16 | 4 |
| | 1422470_at | Bnip3 | BCL2/adenovirus E1B 19 kDa protein-interac ng protein 3; | 3.71E-02 | 224.54 | 954.42 | 4 |
| | 1439148_a_at | Pfkl | ATP-dependent 6-phosphofructokinase, liver type ; | 3.75E-02 | 390.51 | 1,579.83 | 4 |
| | 1449324_at | Ero1l | ERO1-like protein alpha; | 4.71E-02 | 651.93 | 2,260.04 | 3 |
| | 1421057_at | Dnase1l3 | Deoxyribonuclease gamma; | 3.71E-02 | 183.88 | 616.78 | 3 |
| | 1452094_at | P4ha1 | Prolyl 4-hydroxylase subunit alpha-1; | 4.71E-02 | 361.28 | 1,168.42 | 3 |
| | 1451895_a_at | Dusp9 | Dual specificity protein phosphatase ; | 3.71E-02 | 319.3 | 951.66 | 3 |
| | 1417864_at | Pgk1 | Phosphoglycerate kinase 1; | 4.71E-02 | 2,606.08 | 7,562.93 | 3 |
| | 1433604_x_at | Aldoa | Fructose-bisphosphate aldolase; | 3.71E-02 | 2,974.82 | 8,013.74 | 3 |
| | 1418129_at | Dhcr24 | Delta(24)-sterol reductase; | 4.71E-02 | 332.98 | 837.46 | 3 |
| | 1429781_s_at | Ccdc39 | Coiled-coil domain-containing protein 39; | 4.30E-02 | 247.12 | 586.02 | 2 |
| Group C | 1448823_at | Cxcl12 | Stromal cell-derived factor 1; | 4.26E-02 | not present | 808.34 | |
| | 1438945_x_at | Gja1 | Gap junc on alpha-1 protein; | 3.71E-02 | not present | 453.34 | |
| | 1448433_a_at | Pcolce | Procollagen C-endopep dase enhancer 1; | 3.71E-02 | not present | 383.29 | |
| | 1452250_a_at | Col6a2 | Collagen alpha-2(VI) chain; | 3.71E-02 | not present | 342.52 | |
| | 1424770_at | Cald1 | H-caldesmon ; | 4.26E-02 | not present | 272.95 | |
| | 1434679_at | Ncan | Neurocan core protein; | 4.71E-02 | not present | 246.65 | |
| | 1449145_a_at | Cav1 | Caveolin-1; | 2.14E-02 | not present | 130.35 | |
| | 1448494_at | Gas1 | Growth arrest-specific protein 1; | 4.12E-02 | not present | 121.73 | |
| | 1425028_a_at | Tpm2 | Tropomyosin beta chain; | 3.75E-02 | not present | 120.12 | |
| | 1416686_at | Plod2 | Procollagen-lysine,2-oxoglutarate 5-dioxygenase 2; | 4.71E-02 | not present | 101.73 | |
| | 1424114_s_at | Lamb1 | Laminin subunit beta-1; | 3.80E-02 | not present | 71.56 | |
| | 1429479_at | Magef1 | Puta ve uncharacterized protein ; | 4.12E-02 | not present | 51.38 | |

S1. Bone marrow memory plasma cells maintain their transcriptional profile and remain functional during in vitro culture. Related to Figure 1. (A) Original fraction of bone marrow from immunized C57BL/6J mice containing 0.4% of CD138++ plasma cells and purity of isolated plasma cells co-expressing BLIMP-1. (B) Ki-67 versus CD138 staining of total bone marrow. (C) Quantification of IgG, IgA and IgM in the supernatant of cultured plasma cells taken at the indicated time points by ELISA (n=1). (D) Expression of CD138 and BLIMP-1 in plasma cells cultured with ST2 in the presence of APRIL and measured on day 1, 3 and 6 of culture. (E) Pearson correlation of the global transcriptome of ex vivo isolated and 3 days cultured plasma cells. (F) Principal component analysis of two individual gene expression analyses of plasma cells ex vivo isolated and after 3 day culture. (G) Mean expression and fold-change of plasma cell specific genes and survival genes in ex vivo and 3 days cultured plasma cells. (H) Transcriptomes of memory plasma cells ex vivo and after 3 days of culture were analyzed for differentially expressed genes. The table shows statistically differentially expressed genes with adjusted p-value < 0.05 and the corresponding mean expression values.

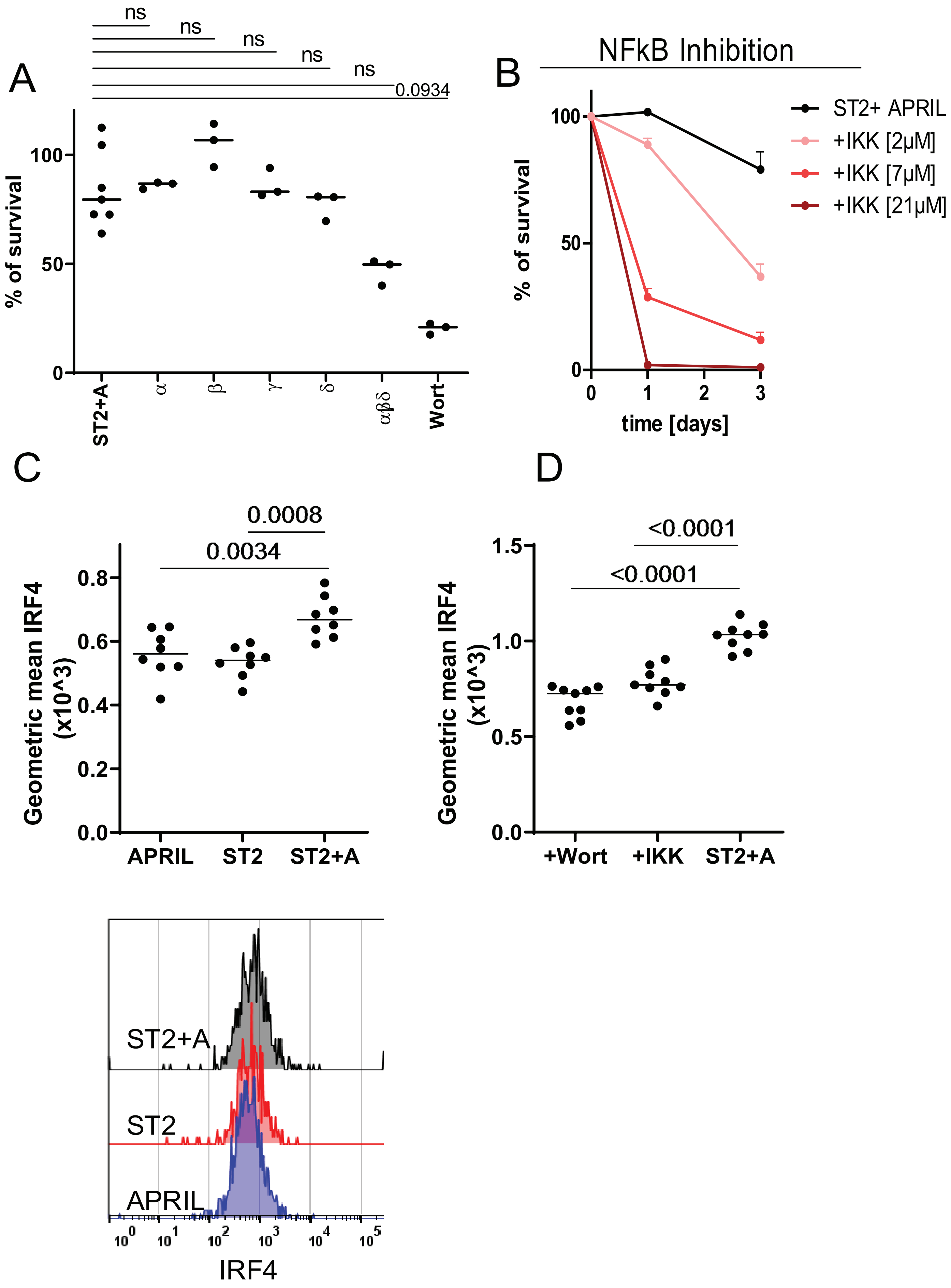


S2. Memory plasma cell survival and IRF4 induction depends on NF-kB and PI3K signaling. Related to Figure 2.
(A) Survival of memory plasma cells treated with inhibitors against different subunits of PI3K or Wortmannin as control (technical replicates n=3-6). Cells were enumerated by flow cytometry on day 1 of culture. Statistics: Kruskal-Wallis test
(B) Survival of memory plasma cells pre-treated with the irreversible inhibitor IKK-16.
Cells were enumerated by flow cytometry on day 1 and 3 of culture.
(C, D) IRF4 protein expression, shown as geometric mean, in CD138+ plasma cells cultured for 1 day with or without ST2 cells, with or without APRIL or pre-treated with either 10 µM Wortmannin or 2.5 µM of IKK16 (pooled from two independent experiments with technical replicates n=6-8 for each group). Statistics: Ordinary one-way ANOVA.

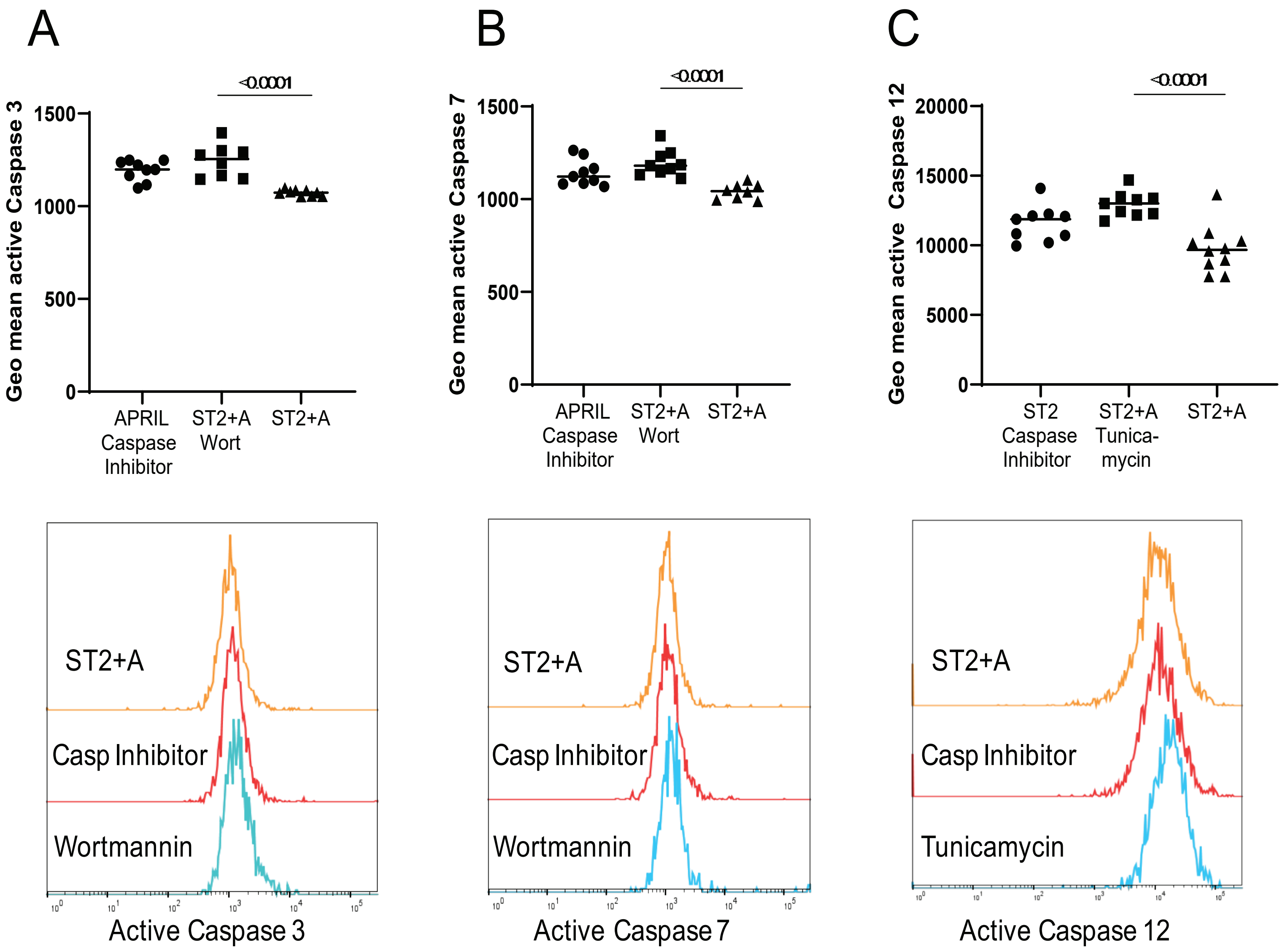


S3. Expression levels of active Caspases 3, 7 and 12 using Wortmannin or tunicamycin as controls. Related to Figure 4.
Expression of active Caspase 3 (A), 7 (B) and 12 (C), shown as geometric mean, in live CD138+ plasma cells cultured for 1 day with APRIL and/or ST2 cells and pan Caspase Inhibitor, and with or without treatment with either 10 µM Wortmannin or 2.5 µM Tunicamcyin for 2 hours (pooled from two independent experiments with technical replicates n=9 for each group). Statistics: Ordinary one-way ANOVA.

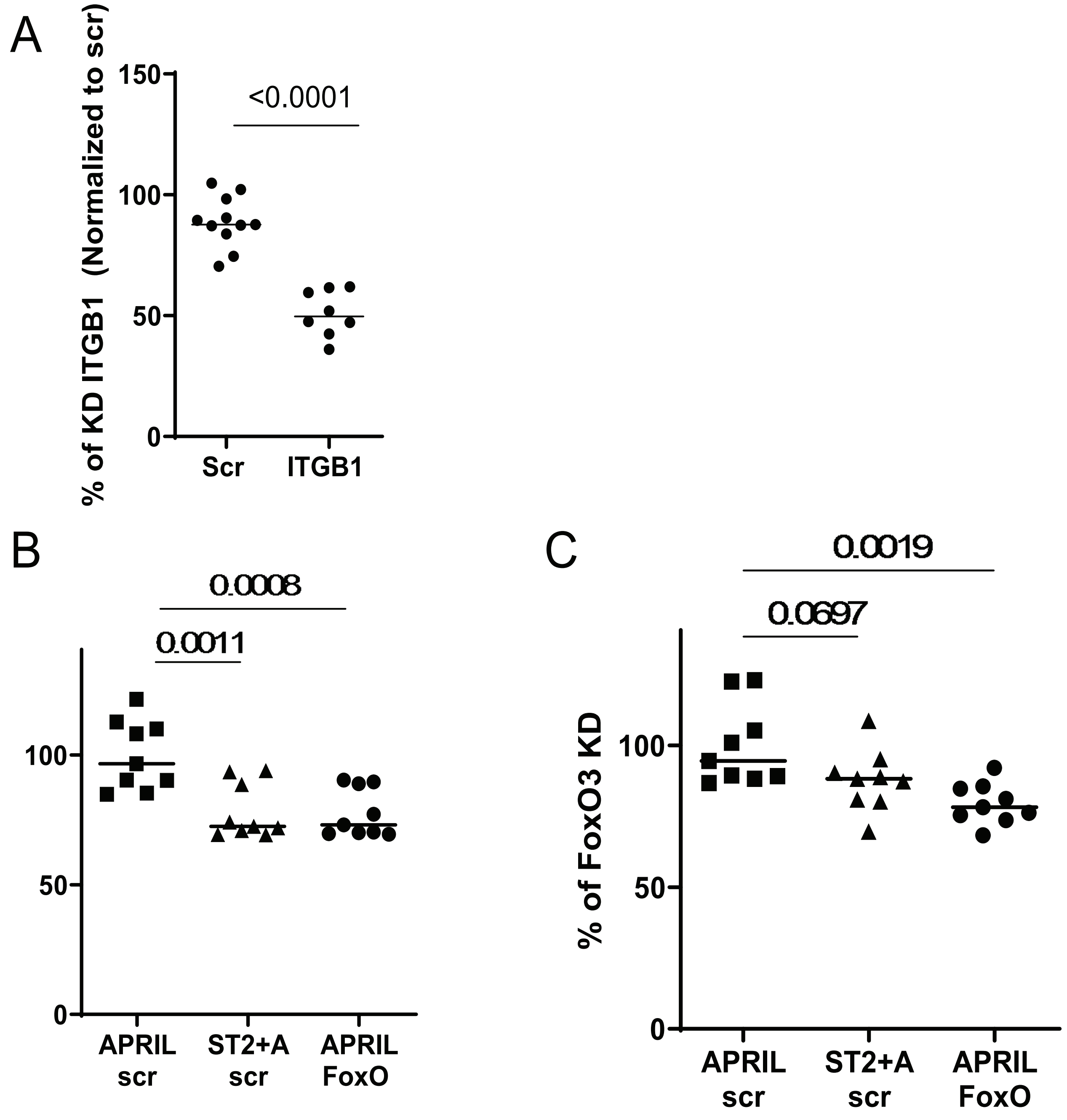


S4. Knock-down efficiency of siRNAs targeting ITGB1, FoxO1 and FoxO3. Related to Figures 1 and 3.
ITGB1 (A), FoxO1 (B) and FoxO3 (C) protein expression was measured in viable CD138+ plasma cells at day 3 of culture with the indicated siRNAs (pooled from three independent experiments with technical replicates n =9 for each group).
Statistics: t-test (ITGB1), ordinary one-way ANOVA (FoxO1 and FoxO3).
